# AP2/ERF Transcription Factor, *Ii049*, Positively Regulates Lignan Biosynthesis in *Isatis indigotica* through Activating Salicylic Acid Signaling and Lignan/Lignin Pathway Genes

**DOI:** 10.3389/fpls.2017.01361

**Published:** 2017-08-04

**Authors:** Ruifang Ma, Ying Xiao, Zongyou Lv, Hexin Tan, Ruibing Chen, Qing Li, Junfeng Chen, Yun Wang, Jun Yin, Lei Zhang, Wansheng Chen

**Affiliations:** ^1^Department of Pharmacy, Changzheng Hospital, Second Military Medical University Shanghai, China; ^2^Department of Pharmaceutical Botany, School of Pharmacy, Second Military Medical University Shanghai, China; ^3^Development and Utilization Key Laboratory of Northeast Plant Materials, School of Traditional Chinese Materia Medica, Shenyang Pharmaceutical University Shenyang, China

**Keywords:** AP2/ERF transcription factor, *Isatis indigotica*, lignan/lignin, metabolic regulation, salicylic acid, secondary metabolism

## Abstract

Lignans, such as lariciresinol and its derivatives, have been identified as effective antiviral ingredients in *Isatis indigotica*. Evidence suggests that the APETALA2/ethylene response factor (AP2/ERF) family might be related to the biosynthesis of lignans in *I. indigotica*. However, the special role played by the AP2/ERF family in the metabolism and its underlying putative mechanism still need to be elucidated. One novel AP2/ERF gene, named *Ii049*, was isolated and characterized from *I. indigotica* in this study. The quantitative real-time PCR analysis revealed that *Ii049* was expressed highest in the root and responded to methyl jasmonate, salicylic acid (SA) and abscisic acid treatments to various degrees. Subcellular localization analysis indicated that *Ii049* protein was localized in the nucleus. Knocking-down the expression of *Ii049* caused a remarkable reduction of lignan/lignin contents and transcript levels of genes involved in the lignan/lignin biosynthetic pathway. *Ii049* bound to the coupled element 1, RAV1AAT and CRTAREHVCBF2 motifs of genes *IiPAL* and *IiCCR*, the key structural genes in the lignan/lignin pathway. Furthermore, *Ii049* was also essential for SA biosynthesis, and SA induced lignan accumulation in *I. indigotica*. Notably, the transgenic *I. indigotica* hairy roots overexpressing *Ii049* showed high expression levels of lignan/lignin biosynthetic genes and SA content, resulting in significant accumulation of lignan/lignin. The best-engineered line (OVX049-10) produced 425.60 μg·g^−1^ lariciresinol, an 8.3-fold increase compared with the wild type production. This study revealed the function of *Ii049* in regulating lignan/lignin biosynthesis, which had the potential to increase the content of valuable lignan/lignin in economically significant medicinal plants.

## Introduction

*Isatis indigotica* Fort. (*I. indigotica*) is a well -known medicinal herb in the family Cruciferae. Its dry root (Ban-Lan-Gen) is frequently used as an anti-inflammatory and antiviral drug for treating hepatitis, influenza, and various kinds of inflammation (Ho and Chang, 2002; Kumagai et al., 2016). Lignans (e.g., pinoresinol, lariciresinol, and their derivatives) in *I. indigotica* have been identified as biologically active ingredients that can significantly inhibit different subtypes of human and avian influenza viruses (Li, 2003; Yang et al., 2013; Li et al., 2015). Additionally, as a typical representative of lignans, lariciresinol also plays an important role in treating cardiovascular diseases and some types of cancer (Adlercreutz et al., 1992; Saarinen et al., 2008). Despite its obvious benefits to human health, the widespread use and availability of lignans are limited by the low concentration (<1‰) in Ban-Lan-Gen (Li, 2003). In this case, improving lignan contents in *I. indigotica* has become an urgent problem to solve.

**Figure 1:**
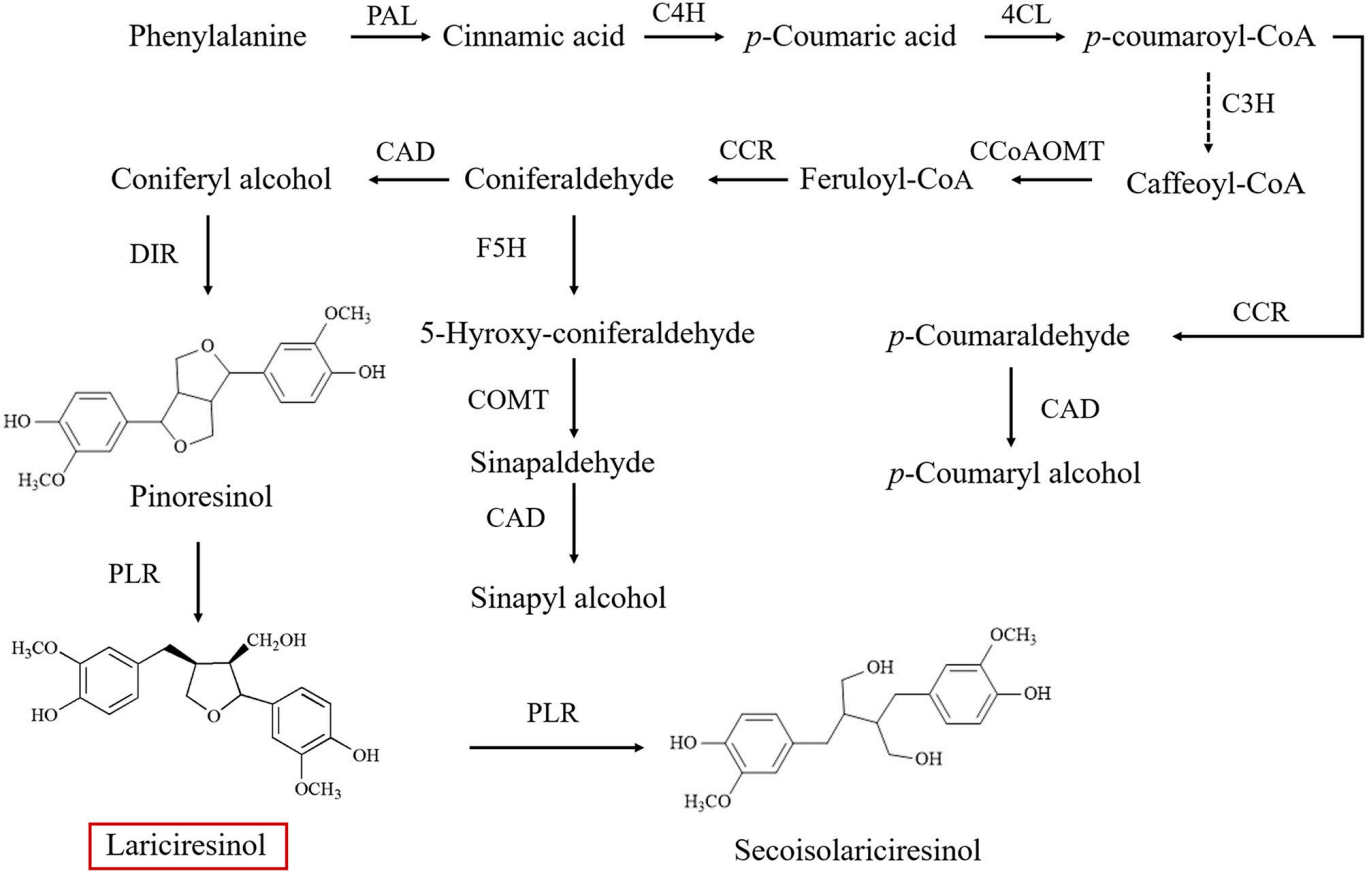
Biosynthetic pathway of lignins in *Isatis indigotica* (PAL, phenylalanine ammonia-lyase; C4H, cinnamic acid 4-hydroxylase; 4CL, 4-coumarate coenzyme A ligase; C3H, coumarate 3-hydroxylase; CCoAOMT, caffeoyl CoA O-methyltransferase; CCR, cinnamoyl-CoA reductase; F5H, ferulate 5-hydroxylase; COMT 1, caffeic acid *O*-methyltransferase; CAD, cinnamyl alcohol dehydrogenase; DIR, dirigent protein; PLR, pinoresinol reductase).

As a subgroup of phenolic compounds, lignans are derived from phenylalanine through the phenylpropanoid pathway (Figure 1; Nakatsubo et al., 2008; Chen et al., 2013; Nguyen et al., 2016). Genes encoding the enzyme cascade of the phenylpropanoid pathway have been widely characterized in *I. indigotica*, including phenylalanine ammonia-lyase (PAL) (Lu et al., 2006), cinnamate-4-hydroxylase (C4H), coumaroyl-CoAligase (4CL) (Zhang L. et al., 2016), coumarate 3-hydroxylase (C3H) (Chen et al., 2015), cinnamoyl-CoA reductase (CCR) (Hu et al., 2011), cinnamyl alcohol dehydrogenase (CAD), dirigent protein (DIR) (Li et al., 2014), and pinoresinol reductase (PLR) (Xiao et al., 2015). An understanding of lignan biosynthesis makes it possible to increase the production of such health-promoting compounds by metabolic engineering. In a previous study, additional expression of *IiPLR* led to 6.3-fold of lariciresinol accumulation in *I. indigotica* hairy roots (Xiao et al., 2015). The overexpression of *IiC3H* resulted in an engineered hairy root producing lariciresinol to an average 1.87-fold of that in the control (Chen et al., 2015). Plant metabolic pathways are known to be complex and usually involve multiple enzymes; thus, increasing the target metabolite production by overexpressing one or two enzymes in the host plant is often not highly efficient (Yu et al., 2012). Transcription factors can channel the metabolic flux by simultaneously regulating the transcription of related biosynthetic genes and manipulating the accumulation of secondary metabolism more obviously (Zhao et al., 2015). However, transcription factors involved in the lignan pathway in *I. indigotica* have not been reported; transcriptional regulation mechanisms of lignan biosynthetic pathway remain unclear.

The APETALA2/ethylene response factor (AP2/ERF) transcriptional factors are characterized by possessing one or two DNA-binding AP2 domains, which possess ~60 conserved amino acid residues (Sakuma et al., 2002; Nakano et al., 2006). This family plays various roles in plant development, stress responses and secondary metabolism in many plant species (Licausi et al., 2013). In recent years, AP2/ERF transcription factors have been reported to play an important role in lignin biosynthesis. For example, *Eriobotrya japonica EjAP2-1* was a novel regulator of fruit lignification via interaction with *EjMYB* transcription factor (Zeng et al., 2015). Other study reported that the overexpression of *Arabidopsis SHINE* (an AP2/ERF gene) in rice showed a 45% reduction in lignin content compared with the wild type (WT) production (Ambavaram et al., 2011). These results suggested that AP2/ERF transcription factors had potential functions in lignin regulation. Whether AP2/ERF transcription factors could regulate the biosynthesis of lignan in *I. indigotica* would be interesting to explore, as lignan shares the same monolignols with lignin (Hano et al., 2006).

In a previous study, methyl jasmonate (MeJA) elicited *I. indigotica* hairy roots were used as resources to identify candidate lignan related AP2/ERF genes. Based on the results of transcriptome and metabolome, four AP2/ERF genes (*Ii007, Ii049, Ii050*, and *Ii080*, which were designated through query of a previously established *I. indigotica* transcriptome inventory by Chen) were identified to be significantly correlated with the biosynthesis of pharmaceutically valuable lignans in *I. indigotica* (Chen et al., 2015). Among these four candidates, *Ii049*, whose homologous gene in *Arabidopsis thaliana* (*At4g13040*) was reported to be a positive regulator for the accumulation of salicylic acid (SA), was an important signaling molecule in the secondary metabolite biosynthesis (Giri et al., 2014). Therefore, the present study, aimed to explore the function of *Ii049* in the regulation of lignan biosynthesis, and also investigate the relationship among *Ii049* expression, SA accumulation and lignan biosynthesis.

## Materials and methods

### Plant material and treatments

*I. indigotica* plants were cultivated in a greenhouse with natural light at 20–25°C in the Second Military Medical University (SMMU), Shanghai, China. Species verification was performed by Professor Hanming Zhang of the School of Pharmacy, SMMU. Seeds of *I. indigotica* were pretreated with 75% alcohol for 5 min, followed by treatment with 0.1% HgCl_2_ for 10 min to obtain sterilized plants. After rinsing five times with sterile distilled water, the sterilized seeds were cultured on the Murashige and Skoog (MS) medium solidified with 0.6% agar for germination. The sterilized plants were grown in a controlled room at 25°C with a 16/8-h light/dark photoperiod cycle and a relative humidity of ~70%. Two-month-old *I. indigotica* plants, with 7–8 leaves, were used for genetic transformation and stress treatments. For stress treatment, plants in sealed boxes were sprayed with 0.1 mM MeJA, SA, and ABA, respectively, until the solution dropped off from the plants. The entire plants were harvested at 0-, 2-, 4-, 6-, 8-, 12-, and 24-h post-treatment. Three independent biological replicates for each group were performed. MeJA, SA, and ABA were purchased from Sigma-Aldrich (USA). All samples were immediately frozen in liquid nitrogen and stored at −80°C until required for quantitative real-time polymerase chain reaction (qRT-PCR) analysis.

### DNA and RNA preparation

Genomic DNA was isolated from 2-month-old *I. indigotica* sterilized plants using the cetyltrimethyl ammonium bromide (CTAB) method (Doyle, 1990). The total RNAs of *I. indigotica* plants were extracted using the RNAiso Plus (Cat. #9108, TaKaRa, Japan). The DNase I (Cat. #GD201-01, Tiangen Biotech Co., China) was used to remove all DNAs from the RNA samples according to the protocol suggested by the manufacturer. The quality and concentration of DNA and RNA samples were examined by ethidium bromide-stained agarose gel electrophoresis and spectrophotometer analysis using a NanoDrop 2000C Spectrophotometer (Thermo Scientific, USA).

### Gene/promoter isolation and analysis

The *Ii049* gene was isolated based on the sequencing result from transcription profiling of *I. indigotica* (Chen et al., 2015) with gene-specific primers *Ii049*-F and *Ii049*-R (Supplementary Table S1). The full-length coding region of *Ii049* was obtained by PCR using the Pfu DNA Polymerase (Cat. #AP221-12, TransGen Biotech Co., China) and the first-strand cDNA as a template. PCR was performed under the following condition: denaturation at 94°C for 1 min, followed by 35 cycles, each one consisting of 94°C for 20 s, 45°C for 20 s, and 72°C for 1 min, followed by a final extension at 72°C for 5 min. Genomic DNA sequence of *Ii049* was obtained by the same reaction system using genomic DNA as the template and the extension time at 72°C in the amplification cycles was prolonged to 3 min. The amplified PCR products were purified and cloned into the PMD18-T vector and then sequenced.

Searching for open reading frame (ORF) and prediction of nucleotide translation products were performed using the ORF Finder tool (https://www.ncbi.nlm.nih.gov/orffinder/). The molecular weight (MW) and theoretical isoelectric point of *Ii049* were predicted using the Vector NTI Advance 11 software. An analysis of protein structure was performed using the Simple Modular Architecture Research Tool (SMART, http://smart.embl-heidelberg.de/). Multiple alignment analysis was performed using the ClustalX software (version 1.80). A phylogenetic tree of *Ii049* and various heterologous AP2/ERF members was constructed using the MEGA5.0 software by the neighbor-joining method (1,000 bootstrap replicates). Gene sequences used for multiple alignment analysis and phylogenetic tree are listed in Supplementary Table S3.

The 5′-upstream region of *IiPAL* was isolated using the Genome Walking Kit (TaKaRa, Japan) according to the manufacturer's instruction with primers *IiPAL*-SP1-R and *IiPAL*-SP2-R. The promoter of *IiCCR* was cloned based on the sequence obtained from Hu with primers *IiCCR*-F and *IiCCR-R* (Hu et al., 2011). All sequences are listed in Supplementary Table S1. PlantCare (http://bioinformatics.psb.ugent.be/webtools/plantcare/html/) and NSITE-PL(http://linux1.softberry.com/berry.phtml?topic=nsitep&group=help&subgroup=promoter) were used to predict the characteristics of the promoters (Lescot et al., 2002; Solovyev et al., 2010).

### Quantitative real-time PCR (qRT-PCR)

The qRT-PCR analysis was performed to determine the transcript abundance of *Ii049*. High quality total RNA (1 μg) was used to prepare the first strand cDNA using the TransScript First-Strand cDNA Synthesis SuperMix Kit (Cat. #AT301-03, TransGen Biotech Co., China) following the manufacturer's protocol. QRT-PCR was performed on a Thermal Cycler Dice Real Time PCR machine (TaKaRa, Japan) using the SYBR-Green PCR Master Mix Kit (Cat. #RR820, TaKaRa, Japan) according to the manufacturer's instruction. The expression levels were normalized with the actin control gene using the 2^−ΔΔCt^ method (Vandesompele et al., 2002; Udvardi et al., 2008). All the primers for qRT-PCR are listed in Supplementary Table S2.

### Subcellular localization in protoplast

The encoding region without the stop codon of *Ii049* was cloned into the *pCAMBIA1301*-GFP vector by *Nco* I and *Spe* I sites to generate *Ii049*-GFP to determine the subcellular localization of *Ii049*. The primers used for subcloning are listed in Supplementary Table S1. The sequence and fusion of GFP under the control of the cauliflower mosaic virus (CaMV) 35S promoter were confirmed by DNA sequencing. Plasmid *Ii049*-GFP was transiently expressed into rice protoplasts using the polyethylene glycol-mediated transformation for the observation of subcellular localization. Transfected protoplasts were incubated overnight at room temperature and the transient expressions were visualized using a confocal laser scanning microscope (Nikon, Japan) as described by Tan et al. (2015).

### Construction of *Ii049* RNAi vector and *I. indigotica* transformation

The RNA interference (RNAi) approach was used to knock down *Ii049* to determine the essential role of *Ii049* in the regulation of lignan biosynthesis in *I. indigotica*. A less conserved region at the C-terminus of *Ii049* (371 bp) was used to interfere with the expression of *Ii049*. Gene specific primers *Ii049*-RNAi-F and *Ii049*-RNAi-R (listed in Supplementary Table S1) were used to amplify the fragment with two restriction sites at both ends. After sequence confirmation, the PCR product was inserted into the modified *pC1300-pHANNIBAL* vector (Tan et al., 2015) upstream and downstream of the pyruvate orthophosphate dikinase (PDK) intron with opposite orientations to generate vector p*Ii049*-RNAi (see Supplementary Figure S3).

### Generation of transgenic hairy root

Hairy root cultures of *I. indigotica* were initiated by infecting the 2-month-old sterilized leaf-disk with *Agrobacterium tumefaciens* strain C58C1 as described in a previous study (Chen et al., 2015; Xiao et al., 2015). Plasmids p*Ii049*-RNAi together with *pC1300-pHANNIBAL* (Control check 1, CK1) were separately introduced into *A. tumefaciens strain* C58C1 for plant transformation. The infected leaf-disk was placed on the surface of 1/2 MS solid medium and supplemented with 500 mg·l^−1^ cefotaxime. When the transgenic hairy roots were 2–4 cm, single roots were isolated from leaves and cultivated on 1/2 MS solid medium containing 300 mg·l^−1^ cefotaxime. The hairy roots were transferred to fresh 1/2 MS solid medium with a gradual decrease in cefotaxime concentrations (300, 100, and 0 mg·l^−1^) every 2 weeks. The hairy root lines generated were screened using 10 mg·L^−1^ hygromycin for antibiotic-resistant roots. p*Ii049*-RNAi was named as Ri049 lines. The hairy root lines generated through transformation with the blank C58C1 were named as WT line. Approximately 100 mg transgene hairy roots that had the same growth rates as WT lines were cultured in a 250 ml shake flask containing 200 ml of fresh liquid hormone-free half-strength MS medium at 110 rpm and 25°C in the dark. Clonal hairy root cultures were routinely subcultured every 9 days and harvested at 45 days. The fresh weights of root tissues were recorded at days 9, 18, 27, 36, and 45 for studying the biomass growth rate. After culturing for 45 days, the hairy roots were harvested for DNA and RNA extraction, phloroglucinol-HCl staining and metabolite analysis.

### Analyses of *Ii049*-RNAi hairy roots

Genomic DNA was extracted from 45 days transgene hairy roots using the CTAB method. Primers JDPDK-F and JDPDK-R based on the sequence of vector *pC1300-pHANNIBAL* were used to detect the presence of inserted *Ii049* fragment in Ri049 lines. The hygromycin resistance gene (*hpt*) and hairy root *rolb* gene were also checked using primers *hpt*-F/*hpt*-R and *rolb*-F/*rolb*-R. All primers are listed in Supplementary Table S1.

The expression levels of genes involved in lignan/lignin biosynthesis were analyzed using qRT-PCR in all positive lines. Primers used for qRT-PCR are listed in Supplementary Table S2. At least three independent control lines were tested in these experiments, and the mean value was shown as the control. For phloroglucinol-HCl staining, fresh hairy roots were immediately immersed for 2 min in 5% phloroglucinol (dissolved in 100% ethanol) and then incubated with concentrated HCl for 10 min.

### Extraction and determination of lignin, lignan, and SA

The extraction and determination of Klason lignin (acid-insoluble) and acid-soluble lignin were performed according to the protocol of Ma (2007). Lignan contents of transgenic *I. indigotica* hairy roots were measured as previously described with slight modification (Xiao et al., 2015). Hairy roots were collected and dried at 45°C until a constant dry weight (DW) was obtained. Dry hairy roots were ground into a fine powder (2-mm mesh). Then, 100 mg of dry power was extracted with 5 mL of methanol under sonication for 30 min. The supernatant was taken and the power was extracted again with 5 ml methanol. The supernatant was combined and further centrifuged at 4,000 rpm for 5 min at room temperature. The extract was diluted with methanol to 10 ml total volume. The extract (2 ml) of hairy root was evaporated to dryness and the residue was dissolved in 1 mL of 15% acetonitrile. The final solution was filtered through a 0.22-μm organic membrane filter prior to analysis.

HPLC/MS/MS was performed on an Agilent 1200 series coupled with an Agilent 6410 Triple Quadrupole Mass Spectrometer and an electrospray ionization source (Agilent, USA). The mobile phase was acetonitrile (Cat.1.00030.4008, # HPLC grade, Merck, Germany, eluent A) and 5 mM ammonium acetate solution (Cat. #218365000, HPLC grade, Merck, Germany, eluent B). The solvent gradient was used as follows: 0–4.00 min, 14% A; 4.00–4.50 min, 50% A, 4.50–8.50, 85% A. The negative-ion mode and multiple reaction monitoring mode for MS analyses were selected, under the following conditions: a flow rate of 0.3 mL·min^−1^, an Agilent ZORBAX SB-C18 (3.5 μm, 100 × 2.1 mm i.d.) at a column temperature of 35°C and injection with 5 μL samples. Characteristic m/z ions were 357→151 for pinoresinol, 359→329 for lariciresinol, 361→165 for secoisolariciresinol and 137→93 for SA. The HPLC/MS/MS data was acquired and processed using the MassHunter Workstation software provided by the manufacturer. All standards were purchased from Sigma-Aldrich (USA).

### Electrophoretic mobility shift assay

The full length coding sequence of *Ii049* was amplified with the primers *Ii049*-pET-F and *Ii049*-pET-R (Supplementary Table S1), and inserted into the expression vector pET32a (Novagen, Denmark) between the *Nco* I and *Hind* III sites for the generation of *Ii049* recombinant protein. The resulting *Ii049* recombinant protein was expressed in *Escherichia coli* strain BL21(DE3) (Novagen, Denmark). *E. coli* cells harboring *Ii049* were broken by an ultrasonic disintegrator (Cat. #BILON-650Y, Shanghai Bilon Instrument Co. Ltd., China) in a crushed-ice bath at an output level of 5 and a 50 duty cycle for 1 min with 0.5 s interval every second. After centrifugation (19,000 × g, 20 min, at 4°CC), the supernatants were applied to a 5 mL nickel column (Bio-Scale Mini Profinity IMAC Cartridges) for *Ii049* recombinant protein purification according to the manufacturer's recommendation (Cat. #732-4612, Bio-Rad Laboratories, USA). Protein integrity was checked by western blotting using primary anti-His-Tag antibody (Cat. #12698, Cell Signaling Technology, USA) and horseradish peroxidase-conjugated goat anti-rabbit secondary antibody (Cat. #7074, Cell Signaling Technology, USA). The triple tandem copies of the coupled element 1 (CE1) motif (5′-TTCCACCGCCGTTCCACCGCCGTTCCACCGCCG-3′), RAV1AAT (RAA) motif (5′-AGCAACATATAAGCAACATATAAGCAACATATA-3′) and CRTAREHVCBF2CBF2 (CBF2) motif (5′-GCCGTCGATGTTGCCGTCGATGTTGCCGTCGATGTT-3′) were labeled with biotin at the 3' end. The specificity of binding was examined by competition with the unlabeled probes (10×, 50×, 100×). Electrophoretic mobility shift assay (EMSA) was carried out according to the protocol included in the EMSA/Gel-Shift Kit (Cat. #GS009, Beyotime Biotechnology, China).

### Yeast one-hybrid assay

According to the results of EMSA, yeast one-hybrid assay (Y1H) was performed to verify physical binding of *Ii049* with target promoters. The coding sequence of *Ii049* was amplified by PCR and inserted into the pGADT7 vector to form the prey using primers *Ii049*-Y1H-F and *Ii049*-Y1H-R (Supplementary Table S1). The triple tandem copies of CE1, RAA and CBF2 were inserted into pHIS2 between *EcoR* I and *Sal* I as baits. The probe sequences were the same as those used in EMSA. The prey and baits were co-transformed into yeast strain AH109. The interactions were examined on the SD/-Leu-Trp-His containing 100 mM 3-amino-1,2,4-triazole (3-AT). Yeast cells carrying the blank pGADT7 and pHIS2 plasmids were used as negative controls. Yeast cells were incubated at 28°C and observed after 3 days.

### Measurement of lignan contents after exogenous SA treatment

The WT hairy roots in the exponential phase (~18 days) were prepared for SA induction. Then, 100 mM SA was added to 200 mL of 1/2 MS liquid medium while untreated hairy roots were used as a control. Hairy roots were harvested at 0, 1, 3, 5, 7, 10, and 12 h after treatment. All samples were immediately frozen in liquid nitrogen and kept at −80°C until RNA extraction and compound analysis by HPLC/MS/MS. All treatments were performed in triplicate.

### Generation and analyses of *Ii049* overexpression hairy roots

A full-length cDNA coding sequence of *Ii049* was cloned into the binary vector PHB-flag using *Bam*H I/*Spe* I restriction sites under the control of two CaMV 35S promoter (for primers *Ii049*-OVX-F and *Ii049*-OVX-R, see Supplementary Table S1) to construct the *Ii049* overexpression vector (*pIi049*-OVX, see Supplementary Figure S3). The construct was transferred into *Agrobacterium* C58C1 and introduced into leaf explant of *I. indigotica*. The plants that carried the PHB-flag vector were used as control (CK2). The analysis of hairy roots was performed following the aforementioned methods.

## Results

### Isolation and sequence analysis of *Ii049*

The full length genomic sequence and cDNA sequence of *Ii049* were isolated from *I. indigotica* by RT-PCR. The full-length *Ii049* was 1,352 bp in size and consisted of five introns and six extrons (Supplementary Figure S1). The ORF of *Ii049* was 684 bp, encoding a putative AP2/ERF protein of 227 amino acids. The predicted protein had a calculated MW of 27.37 kDa with an isoelectric point of 9.37. Amino acid analysis indicated that the putative protein contained a conserved 58-residue AP2 domain at the 121st-178th amino acids. As MRG and HLG in the AP2 domain, the gene was deemed to be the member of Soloist subfamily (Zhuang et al., 2009), a subfamily of AP2/ERF transcription factors.

**Figure 2:**
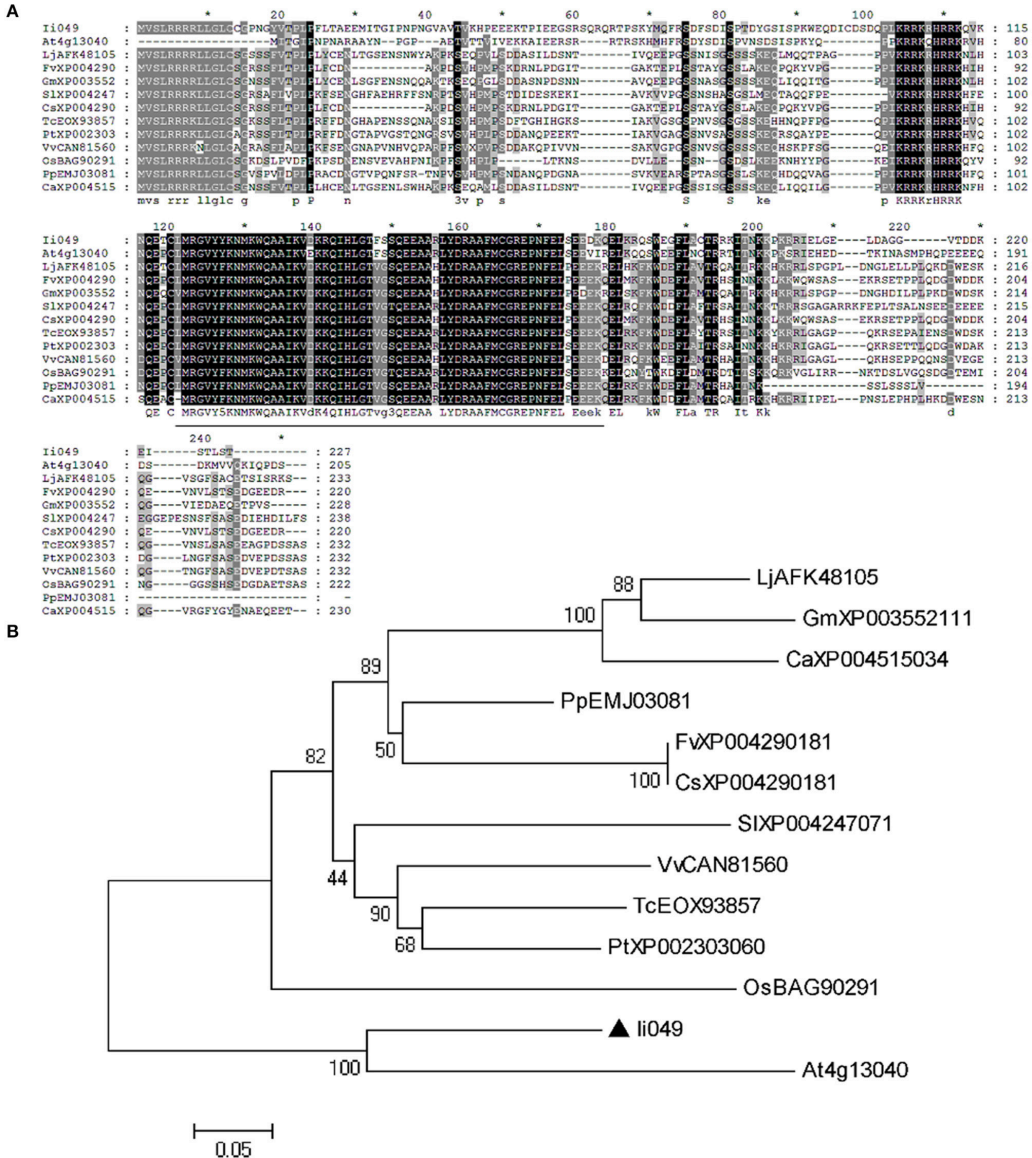
Sequence comparison between *Ii049* and related AP2/ERF family protein. **(A)** Amino acid alignment of *Ii049* with several Soloist-like proteins in different plant species, the AP2 domain has been underlined. **(B)** Phylogenetic tree of *Ii049* and other plant Soloist-like proteins using MEGA 5.0 software based on the neighbor-joining method. Gene names with their accession numbers are listed in Supplementary Table S2.

Sequence alignment was used to further analyze the structural relationship of *Ii049* and other Soloist-like proteins from various plant species. The AP2 domain was highly conserved as it shared an 84–93% amino acid identity with other members of Soloist-like proteins from different species (Figure 2A). Moreover, the phylogenetic analysis showed that *Ii049* was structurally closely related to *AT4g13040* in *Arabidopsis* (Figure 2B). As both *I. indigotica* and *Arabidopsis* belonged to Cruciferae family, *Ii049* and *AT4g13040* might have similar biological functions.

### Expression and induction patterns of *Ii049*

**Figure 3:**
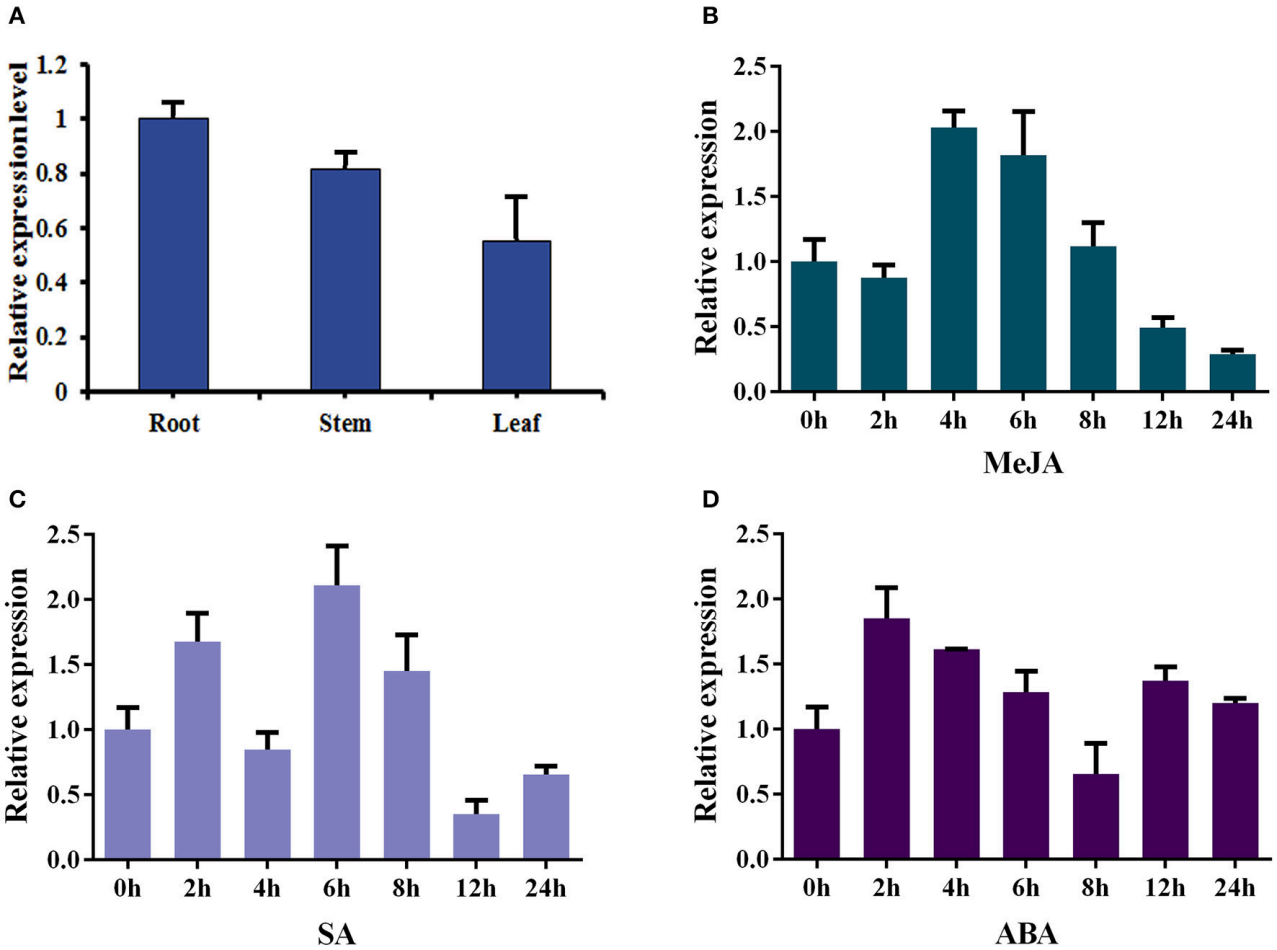
Expression profiles of *Ii049* in different *I. indigotica* tissues **(A)** and under the induction of phytohormones [MeJA **(B)**, SA **(C)**, and ABA **(D)**].

The total RNAs were isolated from roots, stems and leaves of 2-month-old sterilized *I. indigotica* plants to analyze the expression pattern of *Ii049*. The transcript of *Ii049* could be detected in the roots, stems, and leaves. The maximum expression was found in the roots followed by stems, and the least expression was found in the leaves (Figure 3A). Next, the transcripts of *Ii049* were detected in response to MeJA, SA and ABA by qRT-PCR. The result showed that the transcript level of *Ii049* was regulated by MeJA, SA and ABA with significant variations depending on the time and/or the phytohormones (Figures 3B–D). After treatment with MeJA, the expression of *Ii049* slightly declined at 2 h, then increased quickly. The high expression of *Ii049* presented during the 4- to 6-h period (Figure 3B). In response to SA, *Ii049* induction peaked at 6 h, with an increase of ~3.11-fold, and subsequently declined (Figure 3C). For the ABA treatment, the transcript level of *Ii049* was reached a maximum at 2 h (1.85-fold) followed by a decline and was up-regulated again after 12 h treatment (Figure 3D).

### Subcellular localization of *Ii049*

**Figure 4:**
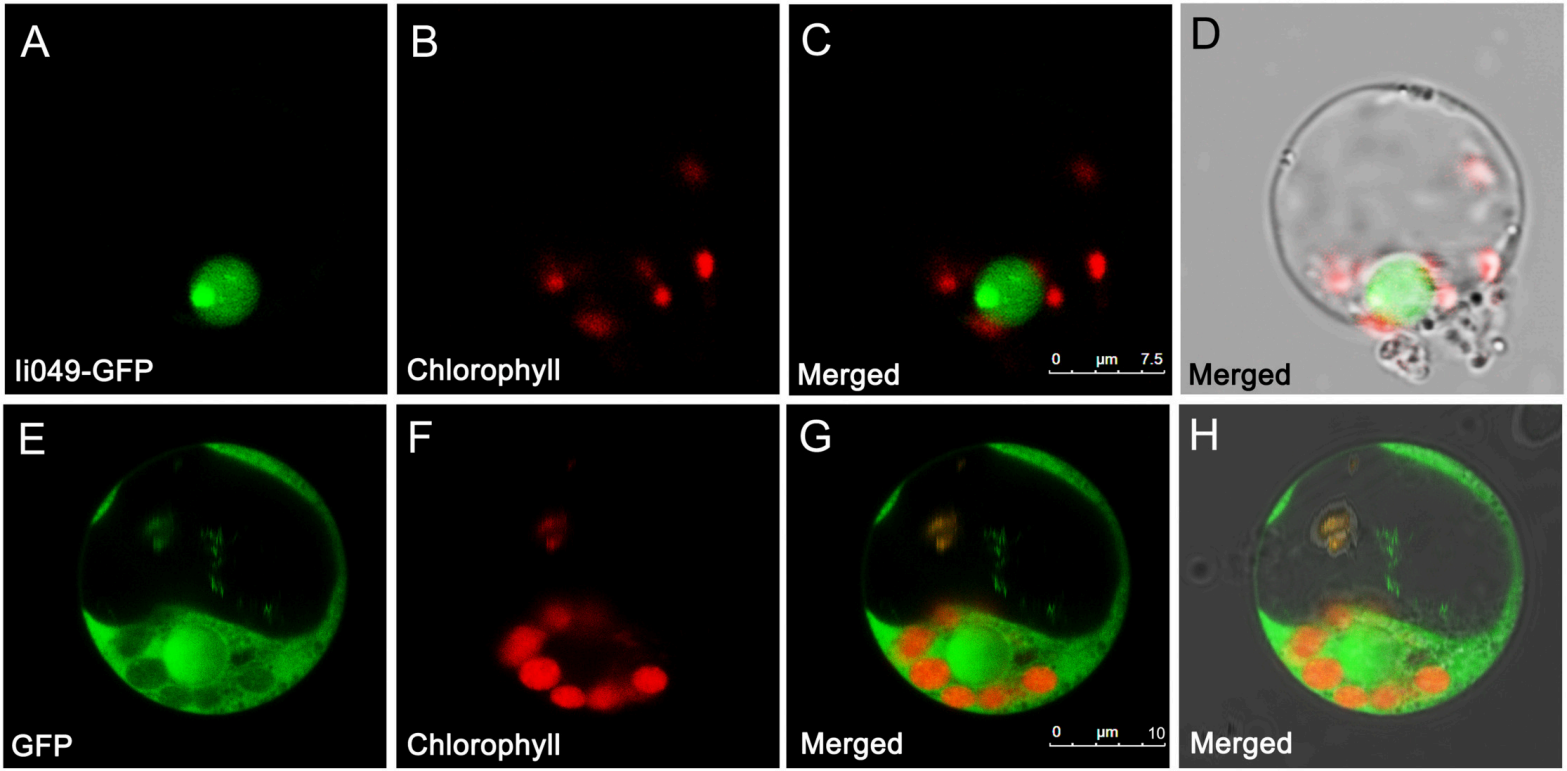
Subcellular localization of *Ii049* in the rice cell. **(A)** A rice protoplast expressing *Ii049*-GFP showing green fluorescent signals in the nucleus. **(B)** The same protoplast cell of **(A)** showing the chlorophyll autofluorescence signal in the plastids. **(C)** The merged signal of **(A,B). (D)** The same protoplast of **(C)** under bright-field. Bars = 7.5 μm. **(E)** A rice protoplast expressing GFP showing green fluorescent signals. **(F)** The same protoplast cell of **(E)** showing the chlorophyll autofluorescence signal in the plastids. **(G)** The merged signal of **(E,F). (H)** The same protoplast of **(G)** under bright-field. Bars = 10 μm. This experiment was repeated three times with similar results.

The subcellular localization of *Ii049* was examined in rice protoplasts using polyethylene glycol-mediated transformation to provide further evidence for the potential role of *Ii049* in transcriptional regulation. The empty vector construct served as control. As shown in Figures 4A–D, the *Ii049*-GFP fusion protein was localized exclusively to the nucleus of the rice cells. In contrast, the free GFP was found in the cytoplasm (Figures 4E–H). Just as expected for a transcription factor, *Ii049* was localized in the nucleus.

### Silencing of *Ii049* affected the production of lignans in *I. indigotica*

**Figure 5:**
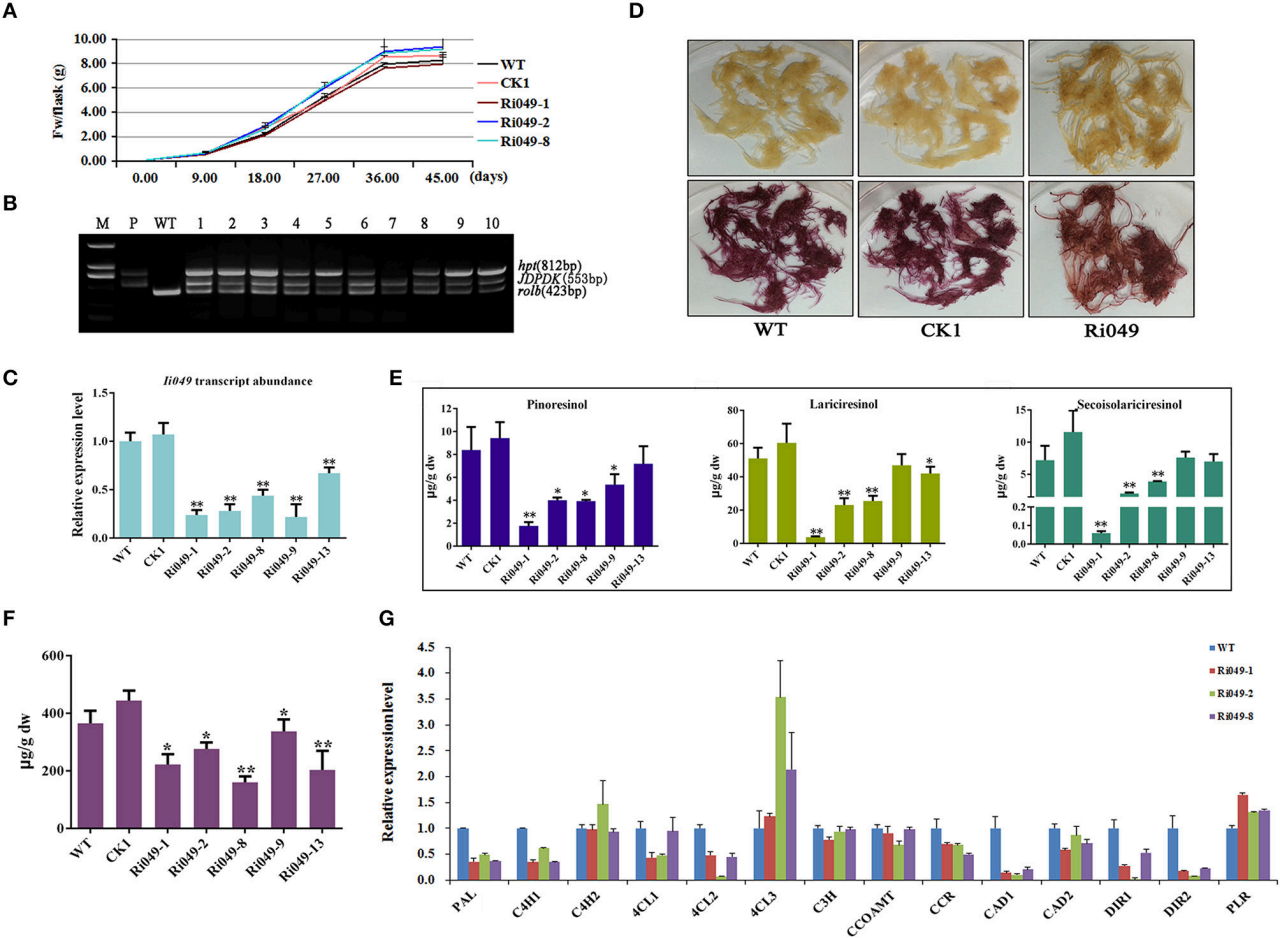
Characterization of transgenic hairy roots silencing *Ii0*49. **(A)** Time course of biomass accumulation of hairy root lines WT, CK1, Ri049-1, Ri049-2, and Ri049-8. **(B)** Representative PCR analyses for the *rolb* and *hpt* genes and the specific gene in transgenic hairy root lines. M, DL-2000 Marker; P, the corresponding engineered bacteria (positive control); and WT, the wild-type hairy root (negative control). **(C)** *Ii049* transcript levels. Bars indicate standard deviation. The level of significance obtained using the Student *t*-test is marked by the following: ^*^P < 0.05, ^**^P < 0.01. **(D)** The phenotype of Ri049 lines before (upper) and after (lower) phloroglucinol-HCl staining. **(E)** HPLC-MS/MS analysis of pinoresinol, lariciresinol and secoisolariciresinol of different Ri049 lines. **(F)** SA content in Ri049 lines. **(G)** Relative expression of lignan/lignin biosynthetic genes in hairy root lines WT, Ri049-1, Ri049-2, and Ri049-8. Each data point is the average of three biological replicates. Bars indicate standard deviation. The level of significance obtained using the Student *t*-test is marked by the following: ^*^P < 0.05, ^**^P < 0.01.

RNAi was used to knock down the expression of *Ii049* to analyze the role of *Ii049* in *I. indigotica*. All types of hairy root lines had the same growth rate and the highest growth rate was at 18 days after inoculation. Although the biomass of each hairy lines had little variations, no significant difference was observed after 45 days (*P* > 0.05) (Figure 5A). The PCR analysis of genomic DNA confirmed the presence of *rolb, hpt* genes and exogenous *Ii049* fragment in Ri049 lines and the absence of exogenous *Ii049* fragment in WT and CK1 lines (Figure 5B). In transgenic plants, five independent lines (Ri049-1, Ri049-2, Ri049-8, Ri049-9, and Ri049-13) with 22–67% observably down-regulated expression of *Ii049* (Figure 5C) were selected for further study.

The hairy roots were stained with phloroglucinol-HCl (Weisner reagent) to reveal any differences of lignan/lignin accumulations between the transgenic lines and WT lines. The color intensity was associated with the presence of lignin and/or wall-bound or secreted phenolic derivatives (Hano et al., 2006). As shown in Figure 5D, WT and CK1 lines presented a similar purple-red color after staining with phloroglucinol-HCl, whereas the Ri049 line displayed a brown-red color after staining, which was weaker than that presented by the WT and CK1 lines. Such a color shift roughly indicated the decrease in lignan/lignin in Ri049 lines (Xiao et al., 2015).

LC-MS/MS was used to confirm further how lignans were modified among the Ri049, WT and CK1 lines. The productions of pinoresinol, lariciresinol and secoisolariciresinol reduced to 21.22–59.71%, 9.51–51.97%, 0.83–67.92%, respectively in lines Ri049-1, Ri049-2, and Ri049-8 (Figure 5E) compared with that in the WT control. Although Ri049-9 and Ri049-13 lines also had lesser lignan contents compared with that in control, no significant difference was found between them (*P* > 0.05).

The homologous gene of *Ii049* in *A. thaliana* plays a role in the SA signaling pathways and is associated with the biosynthesis of SA (Giri et al., 2014). SA contents in transgenic hairy roots were measured to test whether *Ii049* was involved in SA biosynthesis in *I. indigotica*. The contents of SA in transgenic hairy roots decreased to 43.76–75.61% of the WT control as predicted (Figure 5F). The reduction of lignan contents was consistent with the decrease in SA content. Moreover, the transcript levels of *IiPAL*, SA biosynthetic pathway gene, in Ri049 lines were reduced to 35.36–48.75% of the control level (Figure 5G). Therefore, the biosynthesis of lignans in *I. indigotica* might be related to the accumulation of SA.

Transcript analyses were performed on the lignan/lignin biosynthesis genes in transgenic hairy roots using qRT-PCR. Ri049-1, Ri049-2 and Ri049-8 were chosen as three biological replicates. The transcript levels of *IiPAL, IiC4H1, Ii4CL1, Ii4CL2, IiCCR, IiCAD1, IiDIR1*, and *IiDIR2* significantly decreased in Ri049 lines. In particular, the expression levels of *IiC4H2, Ii4CL3*, and *IiPLR* were up-regulated while *IiC3H, IiCCOAMT*, and *IiCAD2* were of the same level as the control (Figure 5G). These results demonstrated that *Ii049* was an important positive regulator in the pathway of lignan biosynthesis, and it might regulate the expression levels of lignan/lignin biosynthetic genes resulting in a change in the lignan contents.

### *Ii049* directly bound to the promoter of *IiPAL* and *IiCCR* in *I. indigotica*

Transcription factors recognize and regulate target genes, which can be measured by EMSA and Y1H (Yu et al., 2012; Tan et al., 2015). Previous reports showed that *IiPAL* and *IiCCR* had the same expression patterns as those of *Ii049*. Both of them had the strongest expression in roots—a pattern where roots of *I. indigotica* (Ban-Lan-Gen) were used as a traditional Chinese medicine. Moreover, a consistent pattern was observed associating the phytohormone induction of lignification and the expression of *Ii049, IiPAL*, and *IiCCR* (Lu et al., 2006; Hu et al., 2011). Based on the reported binding sites of AP2/ERF transcription factor (Wu et al., 2007; Zhu et al., 2010; Yu et al., 2012), CE1 and RAA motifs were found in *IiPAL* promoter (814 bp, Supplementary Figure S2), and CE1, RAA, and CBF2 motifs in *IiCCR* promoter (GenBank accession no. HM636437.1), which were the preferred core binding sites of the AP2/ERF family.

**Figure 6:**
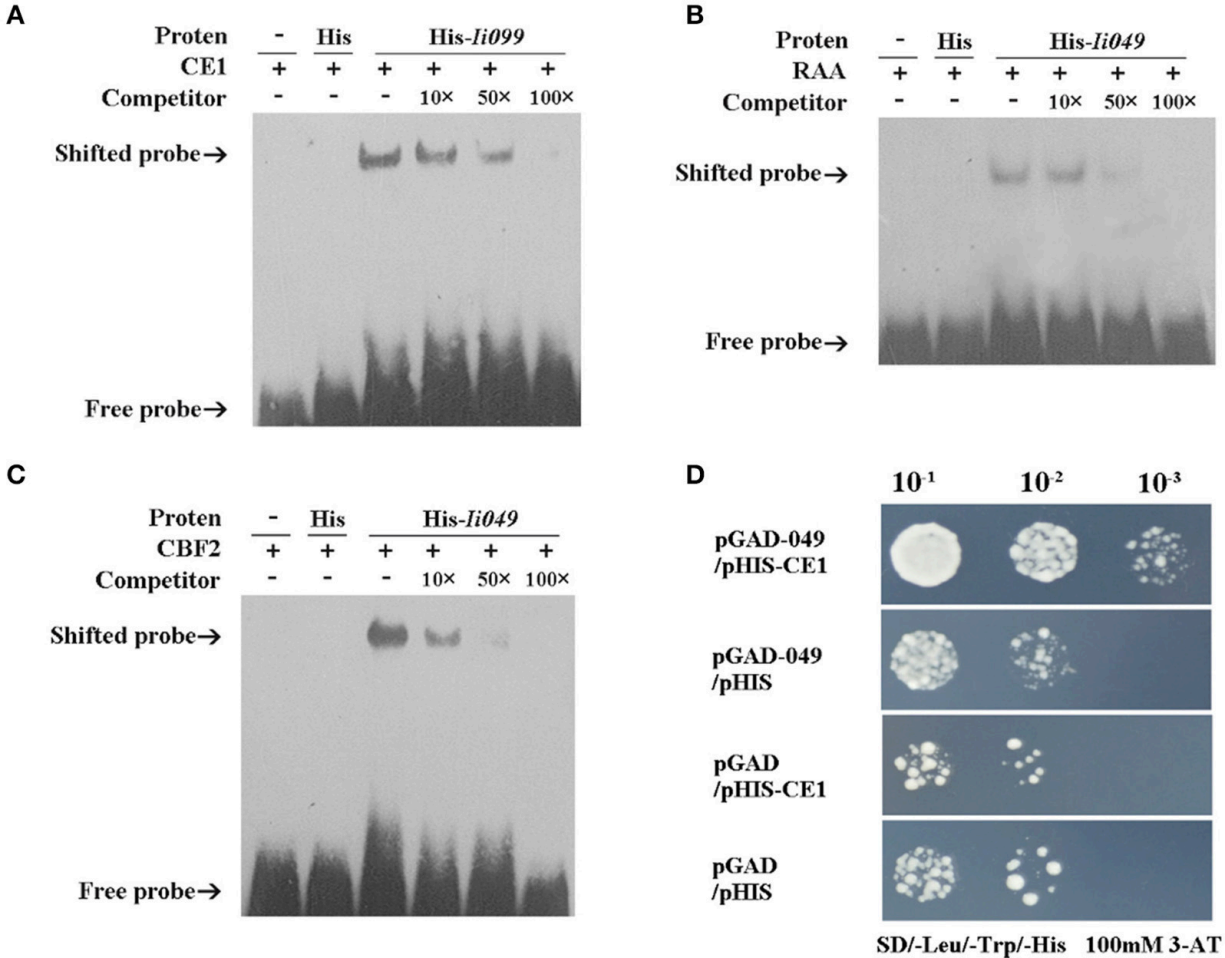
*Ii049* protein bound to CE1, RAA, and CBF2 motifs. **(A–C)** The EMSA assay of *Ii049* protein using biotinylated double-stranded CE1 **(A)**, RAA **(B)**, and CBF2 **(C)** probes, respectively. The detection of probes after reaction with His protein was taken as a negative control. Then, 10 μg of biotin-labeled were incubated with fusion protein at 25°C for 20 min to compete with cold probes (0, 10, 50, 100×) and then analyzed using EMSA. The bands were clarified with a solid black arrow. **(D)** Y1H assay for the interaction between His-*Ii049* with CE1 motif. Triple CE1 motifs were used as bait. Yeast cells carrying pGADT7-*Ii049* and pHIS2-CE1 grew normally on SD-Trp-Ura-His with 100 mM of 3-AT when diluted 1,000 times. Blank pGADT7 and PHIS2 were used as negative controls.

EMSA was performed using the purified recombinant *Ii049* protein (Supplementary Figure S4) and biotin-modified promoter fragments containing three repeated CE1, RAA, and CBF2 motifs, respectively. As presented in Figures 6A–C, the migration of biotinylated probe coding for CE1, RAA, and CBF2 motifs was retarded in the presence of *Ii049* recombinant protein, whereas the His control was not. A competition assay was performed to further investigate the specific binding. The binding activity decreased along with the increase of competitor and when the ratio of the unlabeled to labeled probes was 100:1, almost all CE1-, RAA-, and CBF2-labeled probe were bound (Figures 6A–C, line 6).

Y1H was further performed to verify the physical binding of *Ii049* and CE1, RAA, and CBF2 motifs. The growth of yeast transformants on SD/-Leu-Trp-His containing 100 mM 3-AT was used to confirm the interactions. Only the combination of pGAD7-*Ii049* and pHIS2-CE1 could have a normal growth when diluted 1,000 times (Figure 6D). The results of pGAD7-*Ii049* and pHIS2-RAA/CBF2 were the same as that of pHIS2-CE1 (data not shown). Taken together, these results suggested that *Ii049* was able to interact with RAA, CE1, and CBF2 elements physically.

### SA-induced changes in the lignan accumulation and transcript profile

**Figure 7:**
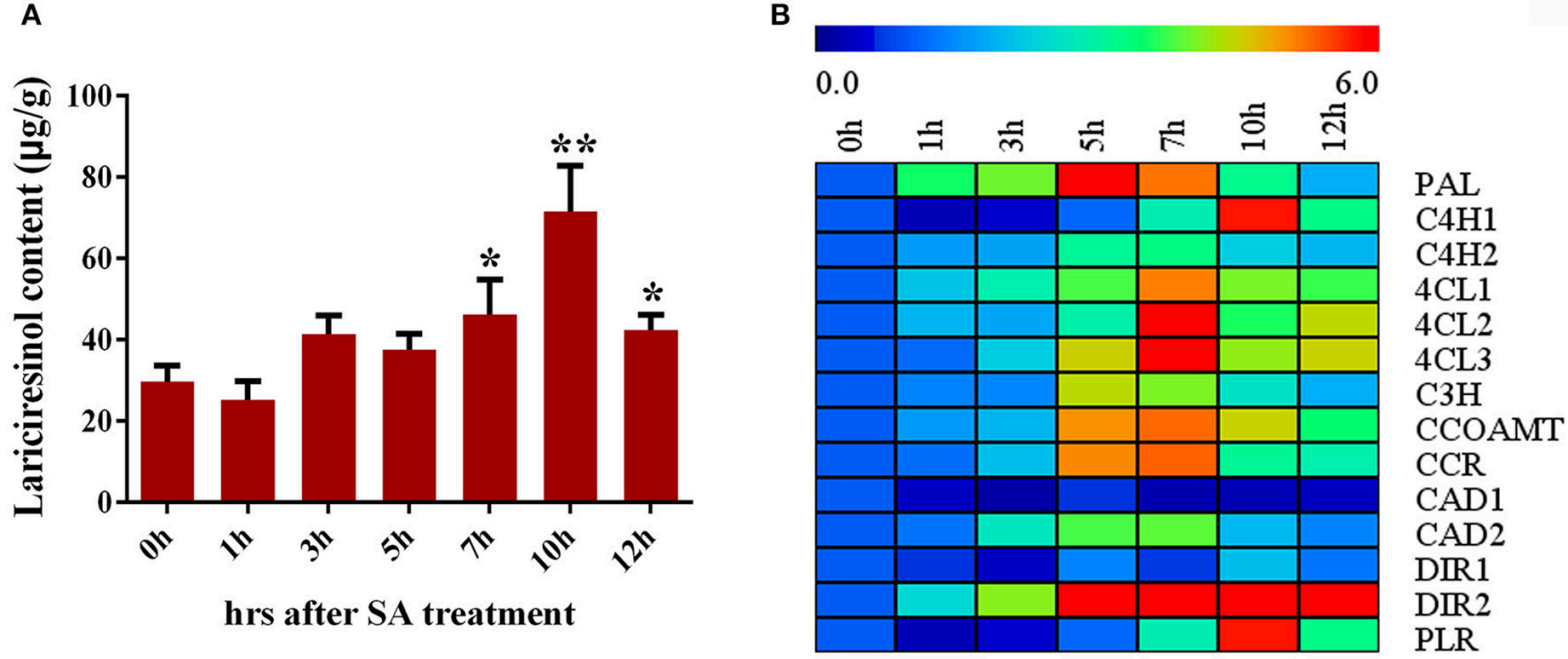
Regulation of SA on lignan biosynthesis in *I. indigotica*. **(A)** Lariciresinol contents under SA treatment (asterisks indicate values that are significantly different) ^*^P < 0.05, ^**^P < 0.01, from 0 h (Student *t*-test). Error bars indicate SD of three biological replicates. **(B)** Relative expression level of lignan biosynthetic genes under SA treatment.

SA signaling is highly important for the synthesis of secondary metabolite. Accumulation of lariciresinol after SA treatment was monitored using LC-MS/MS in *I. indigotica*. As shown in Figure 7A, the content of lariciresinol significantly (*P* < 0.05) increased at 7 h after the treatment and the maximum contents (about 2.41-fold of the control) were observed at 10 h post-inoculation, followed by a gradual decline. Consequently, the genes encoding enzymes of the lignan/lignin biosynthetic pathway were further analyzed. All detected genes had a significant improvement in the transcription level when subjected to SA treatment except *IiCAD1* (Figure 7B). The expression of *IiPAL* increased rapidly and peaked at 5 h. The transcription values of *IiC4H2, Ii4CL1, Ii4CL2, Ii4CL3, IiC3H, IiCCOAMT, IiCCR, IiCAD2*, and *IiDIR2* gradually stimulated and reached the highest level at 7 h, followed by a reduction. The transcript levels of *IiC4H1, IiDIR1*, and *IiPLR* peaked at 10 h after SA treatment. These results indicated SA might play a role in the elicitation process and/or the signal transduction leading to gene activation and finally to the accumulation of lignan in *I. indigotica*.

### Overexpression of *Ii049* improved lignan contents

**Figure 8:**
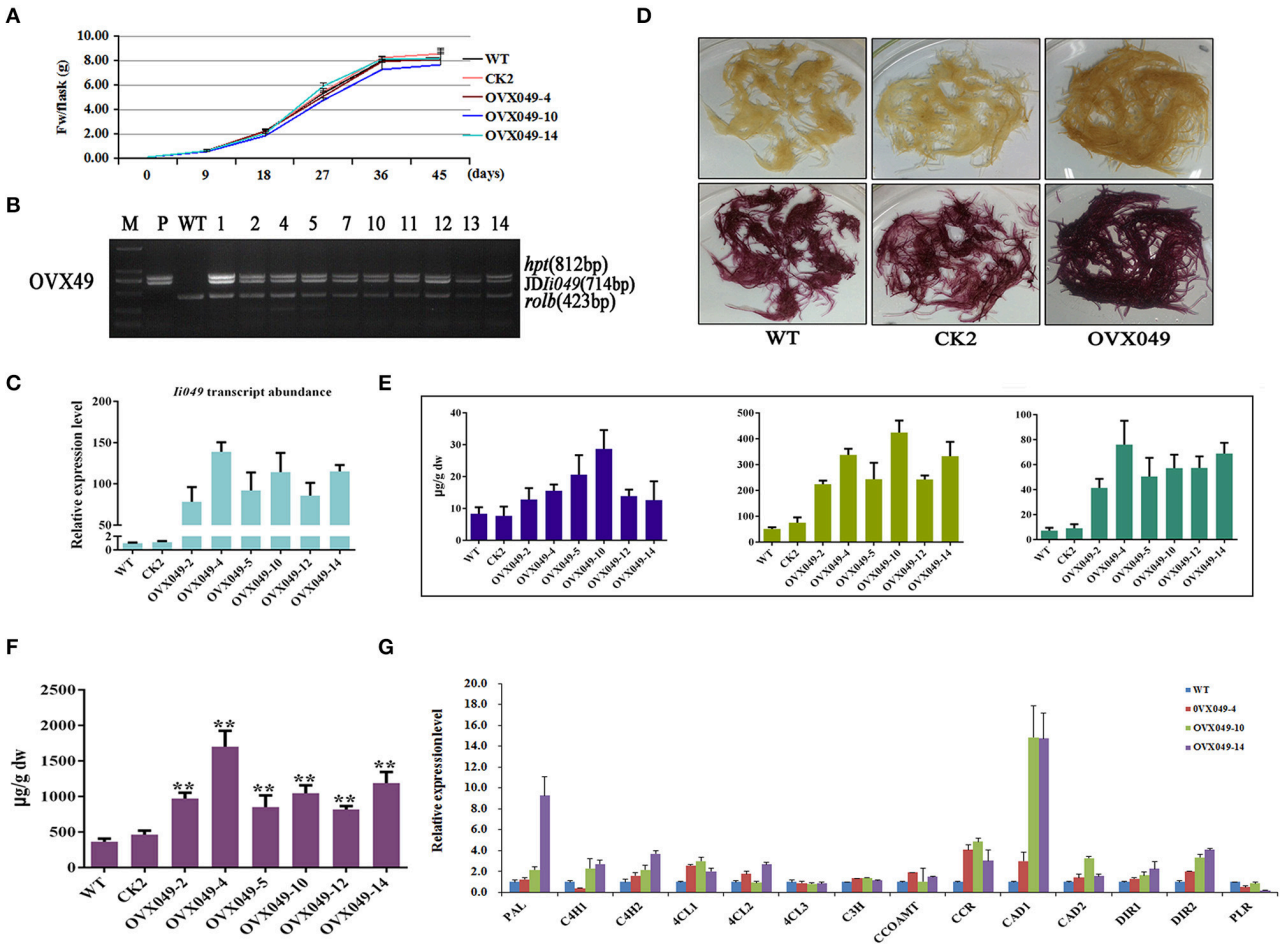
Characterization of transgenic hairy roots overexpressing *Ii049*. **(A)** Time course of biomass accumulation of hairy root lines WT, CK2, OVX049-4, OVX049-10 and OVX049-14. **(B)** Representative PCR analyses for the *rolb* and *hpt* genes and the specific gene in transgenic hairy root lines. M, DL-2000 Marker; P, the corresponding engineered bacteria (positive control); and WT, the wild-type hairy root (negative control). **(C)** *Ii049* transcript levels. Bars indicate standard deviation. The level of significance obtained using the Student *t*-test is marked by the following: ^*^P < 0.05, ^**^P < 0.01. **(D)** Phenotype of OVX049 lines before (upper) and after (lower) phloroglucinol-HCl staining. **(E)** HPLC-MS/MS analysis of pinoresinol, lariciresinol and secoisolariciresinol of different OVX049 lines. **(F)** SA content in OVX049 lines. **(G)** Relative expression of lignan/lignin biosynthetic genes in hairy root lines WT, OVX049-4, OVX049-10, and OVX049-14. Each data point is the average of three biological replicates. Bars indicate standard deviation. The level of significance obtained using the Student *t*-test is marked by the following: ^*^P < 0.05, ^**^P < 0.01.

*Ii049* driven by double CaMV 35S promoter in vector PHB-flag was introduced into *I. indigotica* by *Agrobacterium* C58C1. Just as Ri049 lines, the morphological and growth rates between overexpression (OVX) lines and control lines did not show any difference (Figure 8A). PCR analyses confirmed the integration of the exogenous *Ii049* gene in the transgenic lines and the absence of exogenous *Ii049* in the WT and positive control lines (Figure 8B). The qRT-PCR analysis was conducted to examine the expression levels of the endogenous *Ii049* in the OVX hairy roots. The transcript levels of *Ii049* in all the independent OVX049 lines were significantly enhanced by 78.18- to 138.98-fold compared with the WT lines (Figure 8C). The expression of genes involved in lignan/lignin and SA biosynthesis remarkably improved, especially *IiPAL* and *IiCCR* (Figure 8G). Moreover, the *Ii049* transcript level elevated lines (OVX049) had more SA accumulation compared with that in WT lines (Figure 8F).

The OVX049 hairy roots presented a black-red color after staining with phloroglucinol-HCl (Figure 8D), which preliminarily predicting that OVX049 hairy roots accumulated more lignan/lignin compared with WT and CK2 lines. In agreement with the speculation derived from phloroglucinol-HCl staining, metabolism analysis showed that the OVX049 lines accumulated lariciresinol, which ranged from 4.41- to 8.32-fold of that in the control. Moreover, the contents of pinoresinol and secoisolariciresinol also significantly improved along with the increase in lariciresinol. The highest concentration was found in the OVX049-10 line, followed by OVX049-4 and OVX049-14 lines, with the mean levels of 425.60, 338.09 and 333.69 μg g^−1^ DW, respectively, for lariciresinol (Figure 8E). In comparison, lariciresinol content in the WT lines was 51.13 μg g^−1^ DW. This finding revealed the potential value of *Ii049* in metabolic engineering of lignan production in *I. indigotica*.

### *Ii049* promoted lignin biosynthesis in *I. indigotica*

The total lignin (Klason lignin and acid-soluble lignin) content in the transgenic lines was detected. In the *Ii049* RNAi lines (Ri049-1, Ri049-2, and Ri049-8), the Klason lignin concentration was 85.48, 65.37, and 74.79% compared with that in WT, and the levels of acid-soluble lignin and total lignin also decreased (Table 1). Meanwhile, a significant increase of lignin was found in the overexpression lines OVX049-2, OVX049-10, and OVX049-14. The contents of Klason lignin and acid-soluble lignin were increased by 23.61, 29.82, and 39.68% and 15.59, 35.07, and 36.45%, respectively (Table 1).

**Table 1:** Lignin contents in the *Ii049* transgenic lines.

**Line**	**Lignin (mg/g DW)**		
	**Klason lignin**	**Acid-soluble lignin**	**Total lignin**
WT	223.50 ± 10.79	49.44 ± 2.02	272.94 ± 12.81
CK1	241.05 ± 22.76	49.59 ± 5.01	290.64 ± 27.77
CK2	224.05 ± 9.66	52.40 ± 1.95	276.45 ± 11.61
Ri049-1	191.05 ± 8.61	45.99 ± 1.98	237.04 ± 35.60
Ri049-2	146.12 ± 7.40	43.13 ± 3.72	189.25 ± 18.90
Ri049-8	167.16 ± 14.88	43.48 ± 1.84	210.64 ± 6.33
OVX049-2	276.27 ± 32.51	57.15 ± 3.09	333.42 ± 35.60
OVX049-10	290.14 ± 14.57	66.78 ± 4.33	356.92 ± 18.90
OVX049-14	312.20 ± 5.02	67.46 ± 1.31	379.66 ± 6.33

*Triplicate samples were analyzed for each line. Date are means ± SDs*.

## Discussion

### Characterization of *Ii049*, an AP2/ERF transcription factor in *I. indigotica*

The AP2/ERF family is one of the largest superfamilies of plant-specific transcription factors (Dong et al., 2015). This family is involved in the control of plant growth and developmental programs, stress responses, and secondary metabolism (Licausi et al., 2013). To date, 112 putative AP2/ERF transcription factors have been identified in the *I. indigotica* transcriptome (Chen et al., 2015). However, none of the AP2/ERF family has been functionally characterized so far in *I. indigotica*. Interestingly, a gene encoding an AP2/ERF factor, namely *Ii049*, belonging to the Soloist subfamily was identified recently, which was a good candidate as a regulator of the lignan pathway. In this study, *Ii049* was further characterized, and its role in lignan biosynthesis was analyzed in this study.

The Soloist subfamilies in *Triticum aestivum* L. (Zhuang et al., 2009) and *Hevea brasiliensis* Muell. Arg. (Du et al., 2013) were expressed in all the tested tissues with different expression levels. In *I. indigotica, Ii049* also could be detected in all of the tissues with the highest expression in roots. This result was an indication that *Ii049* might be positively correlated with the synthesis of lignans, as root is the main organ for the accumulation of lignans in *I. indigotica* (Chen et al., 2013).

The AP2/ERF family participates in varieties of signal transduction and is dramatically induced when subjected to environmental stress (Moffat et al., 2012; Cheng et al., 2013; Licausi et al., 2013; Zhang et al., 2015). Defense-related signaling molecules MeJA, SA, and ABA were chosen to examine the expression profile of *Ii049*. The results showed that the expression of *Ii049* was up-regulated in response to MeJA, SA, and ABA. *Ii049* was significantly induced by MeJA just like *AaERF1* and *AaERF2* in *Artemisia annua. Ii049* was also proposed to play a major role in the regulation of secondary metabolism like *AaERF1* and *AaERF2* (Yu et al., 2012). Compared with MeJA and ABA, SA was shown to be the most effective in up-regulating *Ii049*. The results were fully consistent with a previous study on *APD1*, which was primarily regulated by SA (Giri et al., 2014). The expression of *Ii049* under ABA treatment was also detected, as SA and ABA functioned cooperative or antagonistic in terms of plant defense (Xu et al., 2013; Alazem et al., 2014). As expected, ABA could effectively elevate the transcription level of *Ii049*, showing that SA and ABA might exert a synergistic action in *I. indigotica*. Taken together, as a transcriptional factor, *Ii049* might play an important role through multiple signaling pathways in *I. indigotica*.

### *Ii049* was a key regulator of lignan biosynthesis in *I. indigotica*

Lignans, such as lariciresinol, pinoresinol and secoisolariciresinol, are major phytoalexins in *I. indigotica*. When the expression of *Ii049* was down-regulated by RNAi, the accumulation of lariciresinol, pinoresinol and secoisolariciresinol were significantly decreased in *I. indigotica* hairy roots compared with the WT lines. Moreover, *Ii049* also could regulate the biosynthesis of SA just like its homologous in *Arabidopsis* (Giri et al., 2014). In addition, qRT-RCR analysis showed *IiPAL, IiC4H1, Ii4CL1, Ii4CL2, IiCCR, IiCAD1, IiDIR1*, and *IiDIR2* involved in lignan/lignin biosynthesis were repressed in RNAi lines (Figure 5G). *IiPAL, IiCAD1, IiC3H, IiCCR*, and *IiDIR1* were indicated to be the most possible genes involved in lignan biosynthesis (Chen et al., 2015). Interesting, *IiPAL* was also the first key enzyme in SA biosynthesis. All of these suggested that *Ii049* might act as a regulator in lignan accumulation by regulating the pathway genes in lignan/lignin biosynthesis and SA biosynthesis.

Both PAL and CCR are key enzymes in the biosynthesis of lignan/lignin monomers and PAL is also a key enzyme in SA biosynthesis. PAL primarily links primary and secondary metabolism by catalyzing the conversion of L-phenylalanine into cinnamic acid, which is also a rate-limiting step of the phenylpropanoid metabolism (Jones, 1984). CCR plays a key regulatory role in lignan/lignin biosynthesis by catalyzing the nicotinamide adenine dinucleotide phosphate-dependent reduction of cinnamoyl-CoA esters to their corresponding cinnamaldehydes (Jones et al., 2001; Hu et al., 2011). The present study also showed that *IiPAL* and *IiCCR* were two of the most possible genes involved in lignan/lignin biosynthesis in *I. indigotica* (Chen et al., 2015).

AP2/ERF family can be divided into five subfamilies: AP2, DREB, ERF, RAV, and Soloist (Sakuma et al., 2002; Nakano et al., 2006). Members of different subfamilies were reported to display distinct DNA-binding activities. Members of the AP2 subfamily could recognize the sequence GCAC (A/G) N (A/T) TCCC (A/G) ANG (C/T) (Krizek, 2003; Yan et al., 2012). Many DREB proteins had been shown to bind to a dehydration-responsive element (DRE)/C-repeat (CRT) element [A/G)CCGAC] (Park et al., 2001; Zhang et al., 2014). The ERF proteins were first isolated as GCC-box binding proteins from tobacco (Ohme-Takagi and Shinshi, 1995). The RAV proteins bound specifically *in vitro* to the CAACA domain, namely RAA motif (Kagaya et al., 1999; Matías-Hernández et al., 2014). However, information about the DNA-binding properties of Soloist proteins is still lacking. Despite the generalization, evidence accumulated over the years have proved that ERF proteins bind not only to the GCC box but also to the DER/CRT, CE1, CBF2, and RAA motifs (Wu et al., 2007; Zhu et al., 2010; Yu et al., 2012) and some DREF proteins have been reported to bind to GCC box element (Wan et al., 2011). Based on these, whether *Ii049* could bind to CE1, RAA, and CBF2 motifs in *IiPAL* and *IiCCR* promoters was verified.

The results of EMSA indicated that *Ii049* could interact with the promoters of *IiPAL* and *IiCCR* by binding to the CE1, RAA, and CBF2 motifs (Figures 6A–C). Similar results had been observed in Y1H assay where *Ii049* interacted with the promoter directly (Figure 6D). These results indicated that the change in lignan contents was due to the presence of *Ii049*-binding sites at the promoter of lignan/lignin and SA biosynthetic pathway genes such as *IiPAL* and *IiCCR*.

### SA activates lignan biosynthesis in *I. indigotica*

The phytohormone SA is a key regulator of plant development and stress responses, including, drought, cold and salinity stress, which are partly achieved by enhancing biosynthesis of secondary metabolites (Rivas-San and Plasencia, 2011; Khan et al., 2015). SA was widely used as an elicitor to improve active compounds in some plants. For example, the foliar sprays of 50 ppm SA could improve the contents of polyphenols, tannins, alkaloid and flavonoid and ameliorate water stress in *Simarouba glauca* (Awate and Gaikwad, 2014). In *S. miltiorrhiza*, SA induced the expression of tanshinone biosynthetic genes, such as *SmIPPI, SmHMGR, SmDXS II, SmGGPPS*, and *SmCPS*, coinciding with the induction of SA on improving the tanshinone production (Hao et al., 2015). In *Vitis vinifera* L, SA induced the accumulation of *PAL* mRNA, a key enzyme in phenylpropanoid metabolism, leading to a significant accumulation of phenolic and the development of thermotolerance (Wen et al., 2008). The accumulation of lignan in *I. indigotica* was also induced by SA treatment. Moreover, the expression of all other key genes in the lignan/lignin biosynthesis pathway significantly increased except for *IiCAD1*. This might explain the increased accumulation of lignan.

### Engineering lignan/lignin biosynthesis in *I. indigotica*

Lignans are pharmaceutically active compounds in *I. indigotica* for anti-virus, anti-inflammation, anti-tumor growth and angiogenesis (Li, 2003; Saarinen et al., 2008; Yang et al., 2013; Li et al., 2015). However, their utilization was limited due to the low yield in the roots of *I. indigotica*. The overexpression of *Ii049* in hairy roots of *I. indigotica* increased the expression of lignan/lignin biosynthesis genes and SA content in this study, thereby improving lignan/lignin accumulation. The average production of lariciresinol was 5.9-fold of that in the control (Figure 8E). The results of this study indicated that the regulation of the expression of *Ii049* was a promising option for increasing the accumulation of lignans. Otherwise, the overexpression of *Ii049* significantly enhanced total lignin content in *I. indigotica* hairy roots, almost 1.22- to 1.39-fold of that in WT (Table 1). This study yielded similar results with the MYB transcription factor, which triggered lignin biosynthesis to 1.21- and 1.29-fold in *Salvia miltiorrhiza* and 1.48-fold of the control in loquat (Zhang et al., 2010; Zhang J. et al., 2016). The research might potentially manipulate the amount of lignin in other plant products based on the needs. Moreover, the transcription level of *IiPLR* decreased in the OVX049 lines, which might be due to the complexity in transcriptional regulation (Figure 8G). The overexpression of *IiPLR* resulted in an engineered *I. indigotica* hairy root ovx-2 producing about 353.5 μg g^−1^ DW of lariciresinol, a 6.3-fold increase compared with the WT production (Xiao et al., 2015). This study proposed that the co-expression of *Ii049* and *IiPLR* might shed new light on substantially elevating lignan levels in *I. indigotica*, which might be used in the large-scale commercial production in the future.

## Conclusions

**Figure 9:**
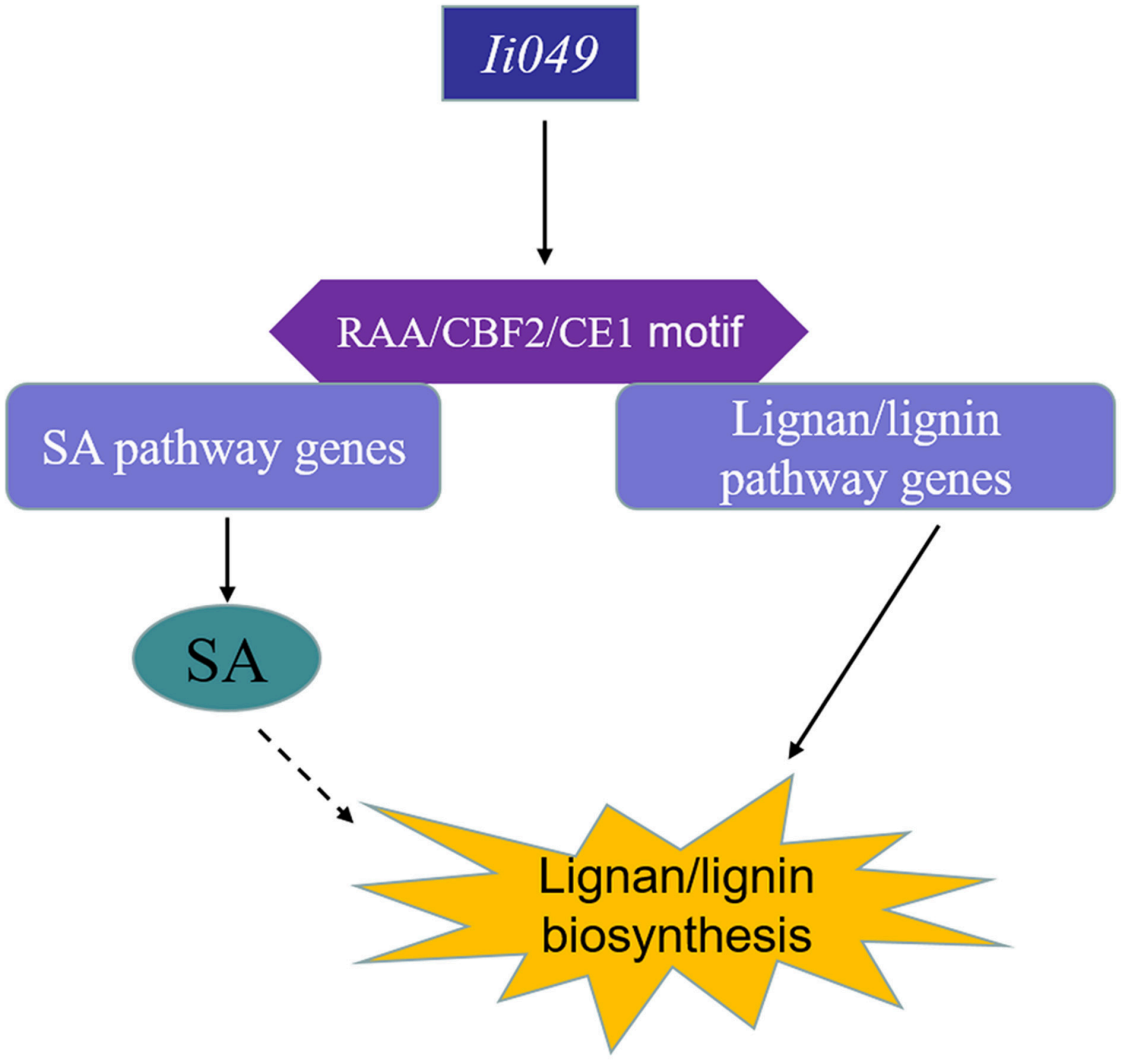
Schematic model of lignan/lignin biosynthesis regulated by *Ii049. Ii049*, an AP2/ERF transcription factor in *I. indigotica*; RAA/CBF2/CE1 motif, cis-element in genes promoter region.

This study showed that *Ii049*, a transcription factor from the AP2/ERF family, acted as a positive regulator of the biosynthesis of lignan/lignin in *I. indigotica. Ii049* was mainly expressed in the roots and specially localized in the nucleus. The contents of lignan/lignin and SA significantly decreased in the RNAi transgenic lines. Moreover, the transcription of lignan/lignin and SA biosynthesis genes was also regulated by *Ii049*. EMSA and Y1H assays showed that *Ii049* might trigger the expression of lignan/lignin and SA pathway genes by binding to CE1, RAA, and CBF2 motifs in their promoters. Also, SA could induce the accumulation of lignan and the expression of lignan/lignin biosynthetic pathway genes. Therefore, *Ii049* controls lignan biosynthesis in two ways: by regulating the genes involved in lignan/lignin biosynthesis and by regulating SA biosynthesis, thus inducing lignan accumulation (Figure 9). In conclusion, this study provided strong evidence for genetic engineering of lignan/lignin production by overexpression AP2/ERF transcription factor.

## Author contributions

RM, YX, JY, LZ, and WC conceived and designed this study. RM, YX, and ZL performed the experiments. HT conducted the experiment of subcellular localization. RM, RC, and QL contributed to data analysis and bioinformatics analysis. JC and YW analyzed the accumulation of compounds through HPLC-MS/MS. RM and YX wrote the final manuscript and all authors read and approved the final version of the manuscript.

### Conflict of interest statement

The authors declare that the research was conducted in the absence of any commercial or financial relationships that could be construed as a potential conflict of interest.

## Supplementary material

Supplementary Figure S1 Nucleotide sequence and the deduced amino acid sequence of *Ii049*.

Supplementary Figure S2 Sequences of *IiPAL* promoter.

Supplementary Figure S3 Schematic diagram of constructed plant expression vectors.

Supplementary Figure S4 The purified recombinant *Ii049* protein was stained with Coomassie Brilliant Blue (lane 1) or visualized after western blotting (lane 2).

Supplementary Table S1 PCR primers used for *Ii049* isolation and vector construction.

Supplementary Table S2 Primers used in qRT-PCR.

Supplementary Table S3 Sequence data for the genes described in this study.

## Abbreviations

4CL: 4-coumarate coenzyme A ligase
ABA: Abscisic acid
C3H: Coumarate 3-hydroxylase
C4H: Cinnamic acid 4-hydroxylase
CAD: Cinnamyl alcohol dehydrogenase
CCoAOMT: Caffeoyl-CoA O-methyltransferase
CCR: Cinnamoyl-CoA reductase
DIR: Dirigent protein
EMSA: Electrophoretic Mobility Shift Assay
MeJA: methyl jasmonate
MS: Murashige and Skoog medium
OVX049: overexpression of *Ii049*
qRT-PCR: quantitative real-time quantitative PCR
Ri049: knockdown the expression of *Ii049*
RNAi: RNA interference
SA: Salicylic acid
WT: Wild-type line.
