# Supplementary figures and images for: AP2/ERF Transcription Factor, Ii049, Positively Regulates Lignan Biosynthesis in Isatis indigotica through Activating Salicylic Acid Signaling and Lignan/Lignin Pathway Genes
Source: Front Plant Sci. 2017 Aug 4;8:1361. doi: 10.3389/fpls.2017.01361 (PMC5543283; doi:10.3389/fpls.2017.01361)

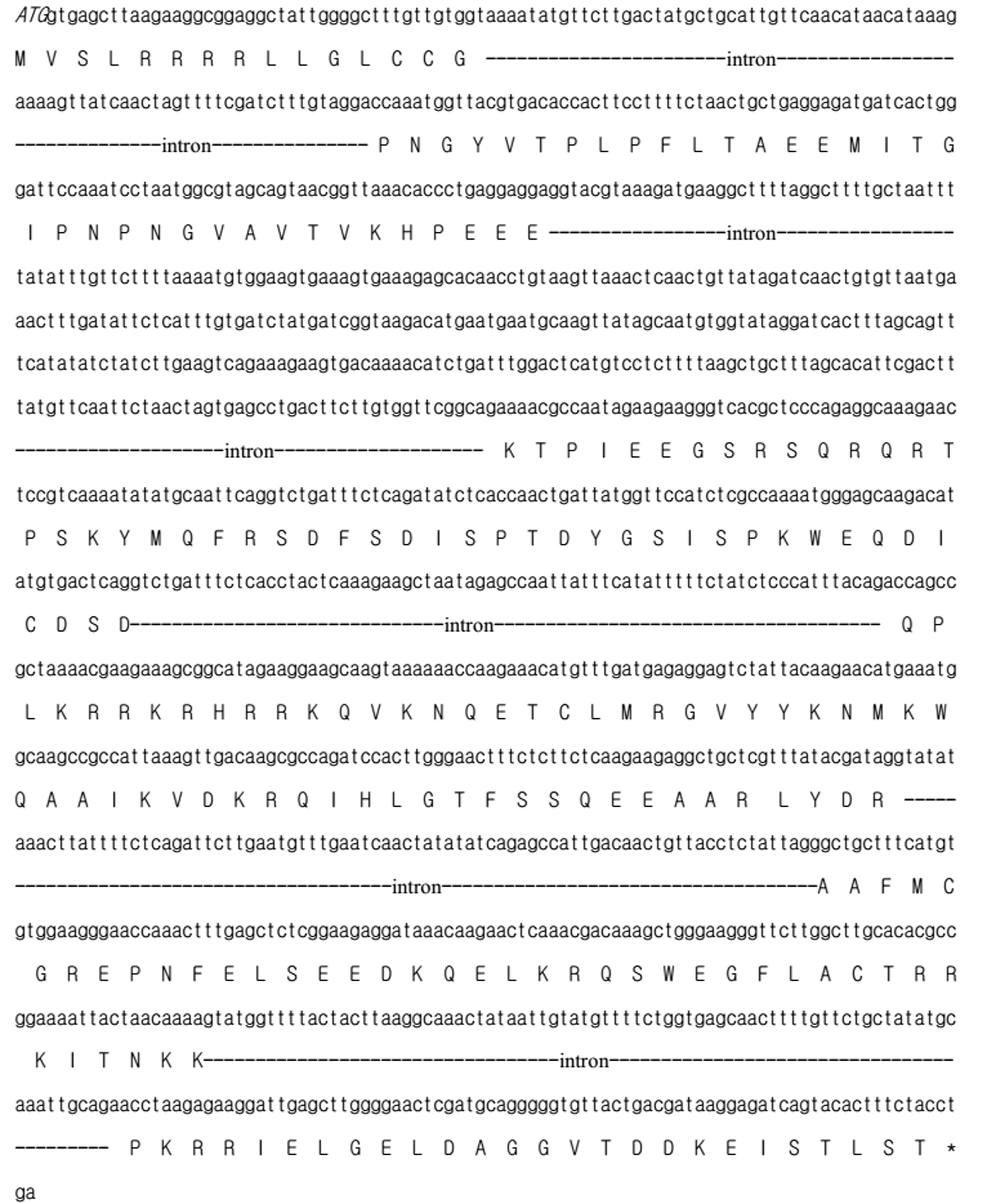

Supplement: Supplementary Figure S1 — Nucleotide sequence and the deduced amino acid sequence of Ii049. [file Image1.TIF]

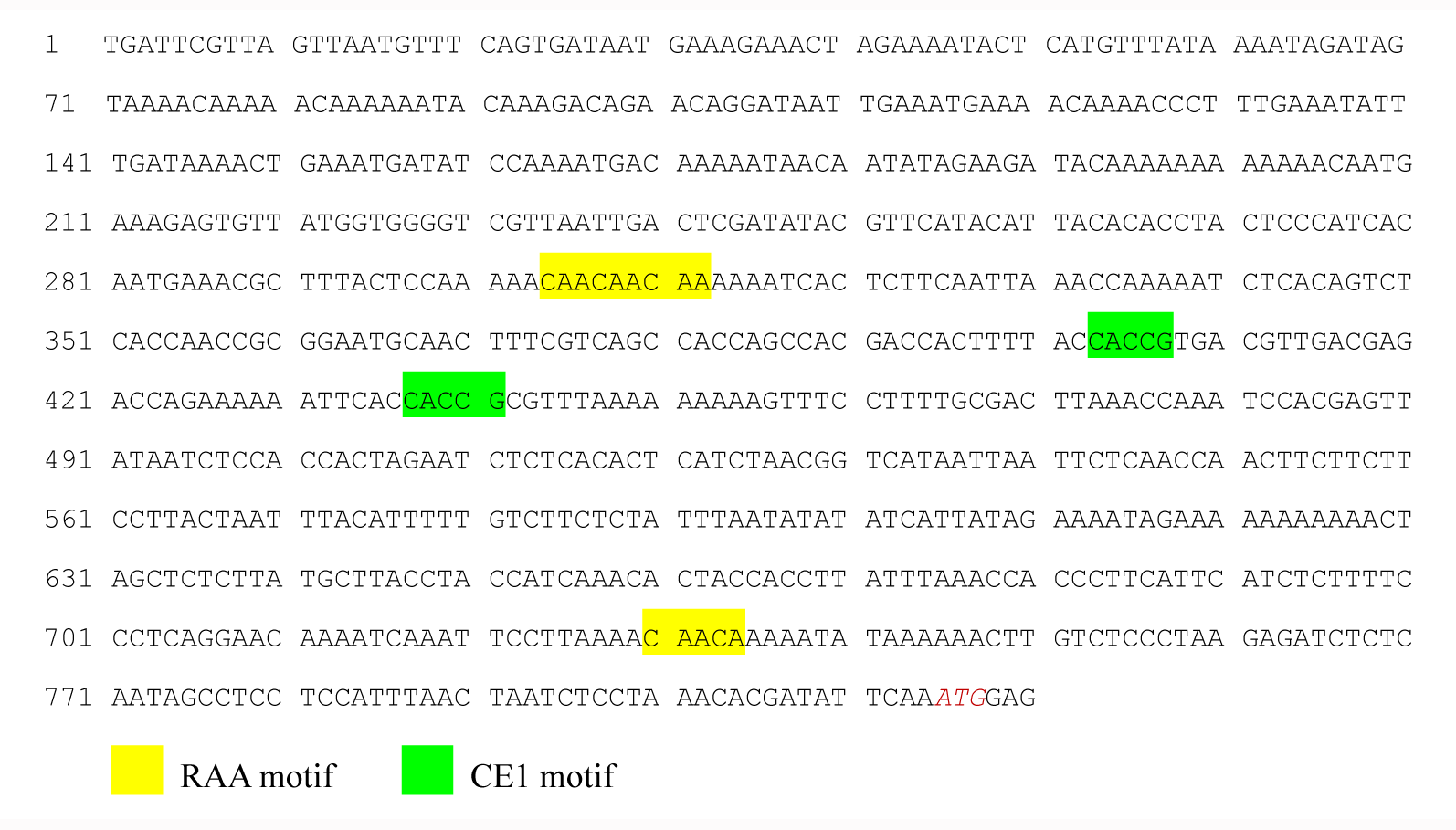

Supplement: Supplementary Figure S2 — Sequences of IiPAL promoter. [file Image2.TIF]

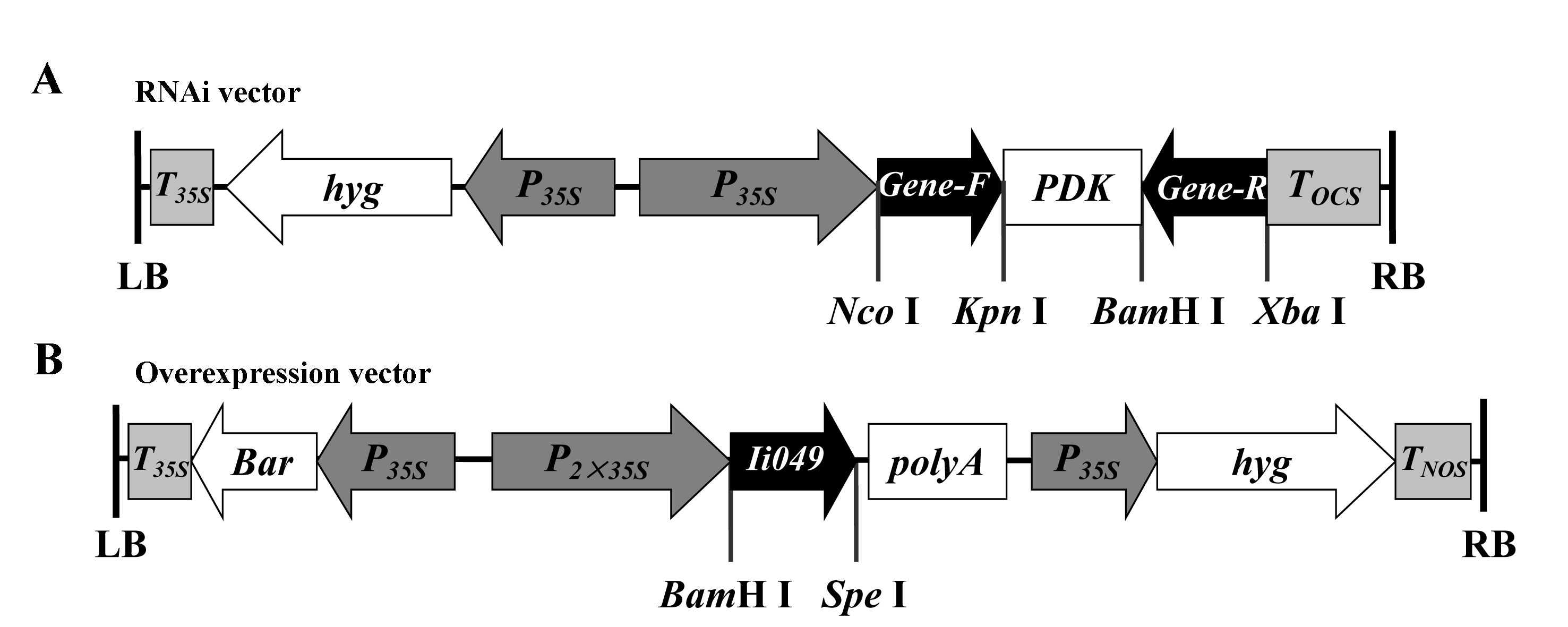

Supplement: Supplementary Figure S3 — Schematic diagram of constructed plant expression vectors. [file Image3.TIF]

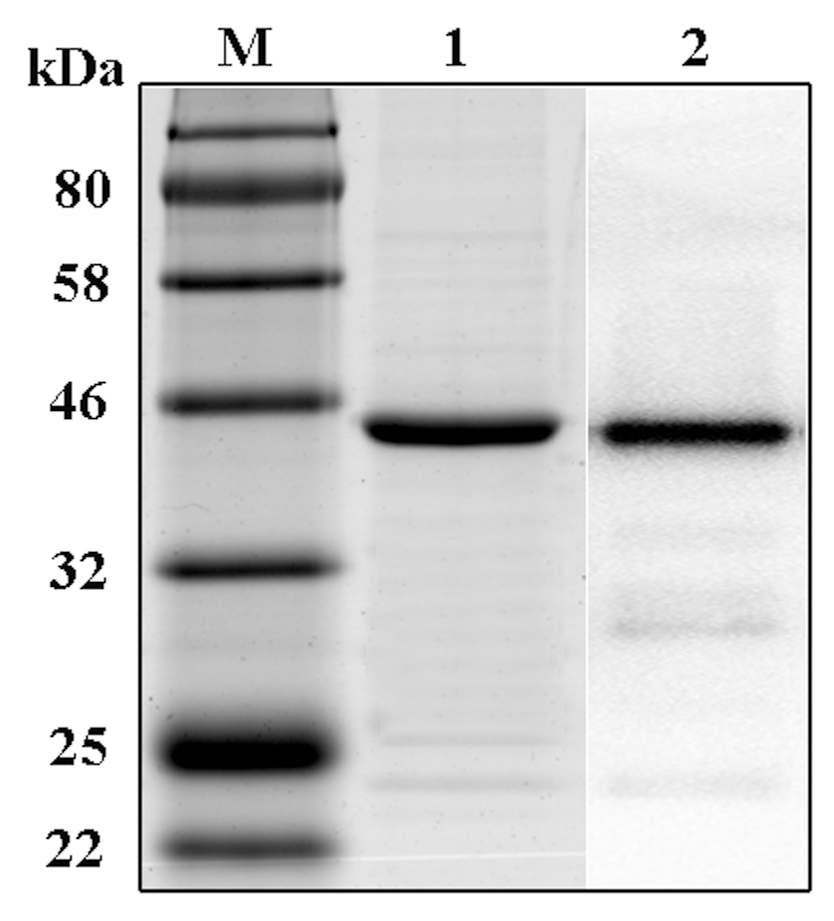

Supplement: Supplementary Figure S4 — The purified recombinant Ii049 protein was stained with Coomassie Brilliant Blue (lane 1) or visualized after western blotting (lane 2). [file Image4.TIF]
