# Supplementary material for: AP2/ERF Transcription Factor, Ii049, Positively Regulates Lignan Biosynthesis in Isatis indigotica through Activating Salicylic Acid Signaling and Lignan/Lignin Pathway Genes
Source: Front Plant Sci. 2017 Aug 4;8:1361. doi: 10.3389/fpls.2017.01361 (PMC5543283; doi:10.3389/fpls.2017.01361)
Supplement: Supplementary Table S1 — PCR primers used for Ii049 isolation and vector construction. [file Table1.DOCX]

| Primer name | Sequence (5'→ 3') |
| --- | --- |
| *Ii049-F* | ATGGTGAGCTTAAGAAGG |
| *Ii049-R* | TCAGGTAGAAAGTGTACTGA |
| *IiPAL-*SP1-R | TCTTCACTTCGTCCAAATGGCTCCCT |
| *IiPAL-*SP2-R | TGTGATGATCCGTTATTCTCCATTTGA |
| *IiCCR*-F | ctggagaagaaatggtgtg |
| *IiCCR*-R | GAGGACTCGTCGACTGGCAT |
| *Ii049*-sub-F | AACCATGGGAATGGTGAGCTTAAGAAGG |
| *Ii049*-sub-R | GGACTAGTGGTAGAAAGTGTACTGATCT |
| *Ii049*-RNAi-F | AAA TCTAGAAAACCATGG ATGGTGAGCTTAAGAAGG |
| *Ii049*-RNAi-R | AAAGGATCCAAA GGTACC CCTCTCATCAAACATGTTTC |
| JDPDK-F | TTGGATTGATTACAGTTGGGA |
| JDPDK-R | GGCGGTAAGGATCTGAGCTA |
| *rolb*-F | GCTCTTGCAGTGCTAGATTT |
| *rolb*-R | GAAGGTGCAAGCTACCTCTC |
| *hpt*-F | CGATTTGTGTACGCCCGACAGTC |
| *hpt*-R | CGATGTAGGAGGGCGTGGATATG |
| *Ii049*-pET-F | AACCATGGGAATGGTGAGCTTAAGAAGG |
| *Ii049*-pET-R | CCAAGCTTGGTAGAAAGTGTACTGATCT |
| *Ii49*-YIH-F | CGGAATTCATGGTGAGCTTAAGAAGG |
| *Ii49*-YIH-R | CCCTCGAGGGTAGAAAGTGTACTGATCT |
| *Ii049*-OVX-F | AAGGATCCATGGTGAGCTTAAGAAGG |
| *Ii049*-OVX-R | CCACTAGTGGTAGAAAGTGTACTGA |
| JD*Ii49*-F | CTCAAGCAAGGATCCATGGTGA |
| JD*Ii049*-R | CGATACCGTCACTAGTGGTAGAA |
