# Supplementary material for: AP2/ERF Transcription Factor, Ii049, Positively Regulates Lignan Biosynthesis in Isatis indigotica through Activating Salicylic Acid Signaling and Lignan/Lignin Pathway Genes
Source: Front Plant Sci. 2017 Aug 4;8:1361. doi: 10.3389/fpls.2017.01361 (PMC5543283; doi:10.3389/fpls.2017.01361)
Supplement: Supplementary Table S2 — Primers used in qRT-PCR. [file Table2.DOCX]

| Primer name | Sequence (5'→ 3') |
| --- | --- |
| *Actin*-F | ATCCTCCGTCTTGACCTTGCT |
| *Actin*-R | TTTCCCGTTCTGCTGTTGTG |
| *Ii049*-qRT-PCR-F | CTCAGACCAGCCGCTAAAAC |
| *Ii049*-qRT-PCR-R | CAAAGTTTGGTTCCCTTCCA |
| *IiPAL*-qRT-PCR-F | AGGAGACGTCGACGCTATGT |
| *IiPAL*-qRT-PCR-R | AGTCGAGATTGCTGCCACTT |
| *IiC4H1*-qRT-PCR-F | CCTGAAGAGTTTAGGCCCGA |
| *IiC4H1*-qRT-PCR-R | AGCTCGAAGTTCTGGACCAA |
| *IiC4H2*-qRT-PCR-F | GTCTCTAATCGCCGTCTTCG |
| *IiC4H2*-qRT-PCR-R | CGCCGAATTTCTTAGCGTAG |
| *Ii4CL1*-qRT-PCR-F | AGACGGCATTTACCGATCAC |
| *Ii4CL1*-qRT-PCR-R | CTAGAGACGGCGGATTTGAG |
| *Ii4CL2*-qRT-PCR-F | CTGAGCTCGGTGAGGATGAT |
| *Ii4CL2*-qRT-PCR-R | CTTTCATCTCGGCGTTCCTG |
| *Ii4CL3*-qRT-PCR-F | GAAGGAGCTTGAAGATGCCG |
| *Ii4CL3*-qRT-PCR-R | CGATCTTCATGTCGGCGTTT |
| *IiC3H*-qRT-PCR-F | GGATATGATCACGGCAGGGA |
| *IiC3H*-qRT-PCR-R | ATCGGTGAGGAAGCATTGGA |
| *IiCCoAOMT*-qRT-PCR-F | ACTCTGTGGAACGGTTCTGT |
| *IiCCoAOMT*-qRT-PCR-R | GATCCGACGGCAGATAGTGA |
| *IiCCR-qRT*-PCR-F | GAGCCAAGCCATACAAGTTC |
| *IiCCR-qRT*-PCR-R | GGCTGGAATCTTGTGATGTC |
| *IiCAD1-qRT*-PCR-F | GGAGGAAAATGGTGGGAGGA |
| *IiCAD1-qRT*-PCR-R | ACGTCAGACTTAGCAAGCCT |
| *IiCAD2-qRT*-PCR-F | GGGAGCTTCATAGGGAGCAT |
| *IiCAD2-qRT*-PCR-R | TCCTTCGACATCAACCACGA |
| *IiDIR1-qRT*-PCR-F | GACTTCTGCTGCCGTTACCAA |
| *IiDIR1-qRT*-PCR-R | GCCACAGGTTCGGATTGATAG |
| *IiDIR2-qRT*-PCR-F | GACTTCAATGCGTGGTTTTGC |
| *IiDIR2*-qRT-PCR-R | TTGTCGGCTCCATCATCAAG |
| *IiPLR*-qRT-PCR-F | GGAGCACGTCTTATCGAAGC |
| *IiPLR*-qRT-PCR-R | GATGTTACCGGCCTCTTTGA |
