# Supplementary material for: AP2/ERF Transcription Factor, Ii049, Positively Regulates Lignan Biosynthesis in Isatis indigotica through Activating Salicylic Acid Signaling and Lignan/Lignin Pathway Genes
Source: Front Plant Sci. 2017 Aug 4;8:1361. doi: 10.3389/fpls.2017.01361 (PMC5543283; doi:10.3389/fpls.2017.01361)
Supplement: Supplementary Table S3 — Sequence data for the genes described in this study. [file Table3.DOCX]

| Name | Protein Accession Numbers in GenBank | Species |
| --- | --- | --- |
| *LjAFK48105* | AFK48105 | *Lotus japonicus* |
| *FvXP004290181* | XP_004290181 | *Fragaria vesca subsp. vesca* |
| *GmXP003552111* | XP_003552111 | *Glycine max* |
| *SlXP004247071* | XP_004247071 | *Solanum lycopersicum* |
| *CsXP004290181* | XP_004290181 | *Fragaria vesca subsp. vesca* |
| *TcEOX93857* | EOX93857 | *Theobroma cacao* |
| *PtXP002303060* | XP_002303060 | *Populus trichocarpa* |
| *VvCAN81560* | CAN81560 | *Vitis vinifera* |
| *OsBAG90291* | BAG90291 | *Oryza sativa Japonica Group* |
| *PpEMJ03081* | EMJ03081 | *Prunus persica* |
| *CaXP004515034* | XP_004515034 | *Cicer arietinum* |
| *AT4g13040* | AY133690.1 | *Arabidopsis thaliana* |
